# Unlocking the Health Potential of Microalgae as Sustainable Sources of Bioactive Compounds

**DOI:** 10.3390/ijms22094383

**Published:** 2021-04-22

**Authors:** Assunta Saide, Kevin A. Martínez, Adrianna Ianora, Chiara Lauritano

**Affiliations:** Marine Biotechnology Department, Stazione Zoologica Anton Dohrn, Villa Comunale, 80121 Napoli, Italy; assunta.saide@szn.it (A.S.); kevin.martinez@szn.it (K.A.M.); adrianna.ianora@szn.it (A.I.)

**Keywords:** microalgae, pharmaceuticals, bioactive molecules, marine biotechnology

## Abstract

Microalgae are known to produce a plethora of compounds derived from the primary and secondary metabolism. Different studies have shown that these compounds may have allelopathic, antimicrobial, and antipredator activities. In addition, in vitro and in vivo screenings have shown that several compounds have interesting bioactivities (such as antioxidant, anti-inflammatory, anticancer, and antimicrobial) for the possible prevention and treatment of human pathologies. Additionally, the enzymatic pathways responsible for the synthesis of these compounds, and the targets and mechanisms of their action have also been investigated for a few species. However, further research is necessary for their full exploitation and possible pharmaceutical and other industrial applications. Here, we review the current knowledge on the chemical characteristics, biological activities, mechanism of action, and the enzymes involved in the synthesis of microalgal metabolites with potential benefits for human health.

## 1. Introduction

Microalgae represent one of the most diverse groups of microorganisms in freshwater and marine systems [1]. Microalgae are eukaryotic organisms that contribute 40% of global productivity [2]. They are characterized by huge variety of species that grow in diverse environments and live in extreme conditions, including high and low temperatures, light intensity, pH, and salinity. Their cultivation is quite simple, with fast growth rates compared to marine plants and macroorganisms. Thanks to their metabolic plasticity, they can trigger the production of several compounds with possible applications in various biotechnology sectors (e.g., food, energy, health, the environment, and biomaterials) [3,4]. Marine microalgae have attracted increasing interest due to the possibility of cultivating them in large quantities in an eco-friendly and eco-sustainable way, thus overcoming the problem of supply for chemical and bioactivity characterization and avoiding disruptive collection practices required for macroorganisms. This is a property of particular significance, considering the rising need for new bioactive compounds for pharmaceutical applications due to the increasing incidence of cancer, infectious diseases, viral infections, antibiotic resistance, and the insurgence of other human pathologies [4].

Different classes of microalgal-derived compounds have been identified and several have shown specific biological activities, such as anticancer [5,6] anti-inflammatory [3,7,8], anti-diabetes [9], antioxidant [10], anti-tuberculosis [11] anti-epilepsy [12], anti-hypertensive [10], anti-atherosclerosis [10], anti-osteoporosis [10], and immunomodulatory activities [13,14]. In addition, various authors have shown that different culturing conditions, including incubation with predators, influence microalgal bioactivities [3,15] (the so-called OSMAC approach: one strain many compounds) triggering the activation of specific metabolic pathways [8,16,17,18,19,20].

**Figure 1:**
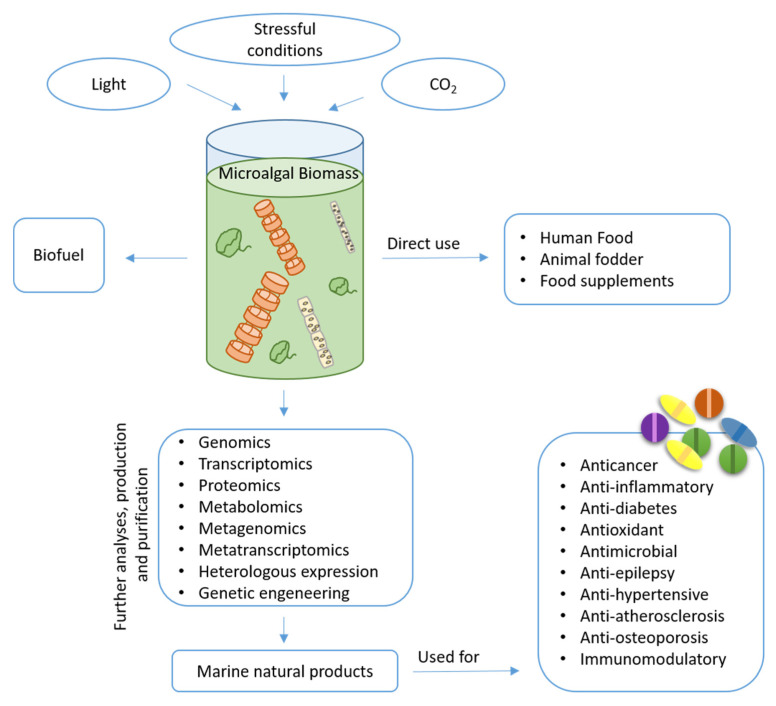
A schematic representation of microalga biomass for different applications.

However, natural products from microalgae remain largely unexplored compared to those obtained from land plants. The identification of bioactive compounds is a complex task that requires multidisciplinary approaches. The continuous upgrading of analytical and molecular techniques is important in this process and is a prerequisite for the targeting of novel products by means of high-throughput strategies [21]. In the last decade, growing public and private interests and investments in marine biotechnology have increased the possibility of generating information and collecting huge amounts of data to enhance a wider understanding of different cellular processes and biological phenomena. Additionally, marine biotechnology makes use of -omics methodologies (such as genomics, transcriptomics, proteomics, metabolomics, metagenomics, and metatranscriptomics) associated to heterologous expression or genetic engineering to identify possible bioactive species and increase the production of the desired products [22]. The number of potential marine natural products (MNPs) isolated currently exceeds 32,000 with hundreds of new compounds being discovered every year [23]. Microalgae are known to be excellent sources of pigments, lipids, vitamins, toxins and other chemicals [24], with possible application in different fields (Figure 1). Here, we discuss their application mainly in the biomedical field, reviewing current knowledge on the isolated compounds.

## 2. Microalgal Bioactive Compounds

### 2.1. Pigments

Microalgae produce a variety of pigments of various color shades and biological activities. These include chlorophylls, carotenoids, xanthophylls, and phycobiliproteins. Recent studies have revealed that these pigments play an important role in the prevention of human disease and the maintenance of good health [25]. Saide et al. [6] also reviewed that chlorophyll degradation products may be active, such as the compound Pheophorbide *a.* Pheophorbide *a* has attracted widespread attention in recent years as a non-invasive and highly selective approach for cancer treatment. The review also reports other important bioactivities shown for Pheophorbide *a*, such as antiviral, anti-inflammatory, antioxidant, immunostimolatory and anti-parasite activities. The biosynthetic carotenoid pigment pathway has been extensively studied. Diatoms show different metabolic features compared to plants [26] and use unique pigments, that are not present in other species, for light harvesting and photoprotection [27]. The biosynthetic carotenoid pathway is still not completely understood, and the reactions and enzymes from violaxanthin to diadinoxanthin are still hypothetical [28]. Lohr and Wilhelm 1999 and Dambek et al. 2012 [29,30] proposed the hypothesized pathway of carotenoid biosynthesis in *Phaeodactylum tricornutum.* However, there is a great interest in increasing carotenoid production and a recent study used genetic transformation of this diatom to increase its carotenoid content [28].

#### 2.1.1. Fucoxanthin

**Figure 2:**
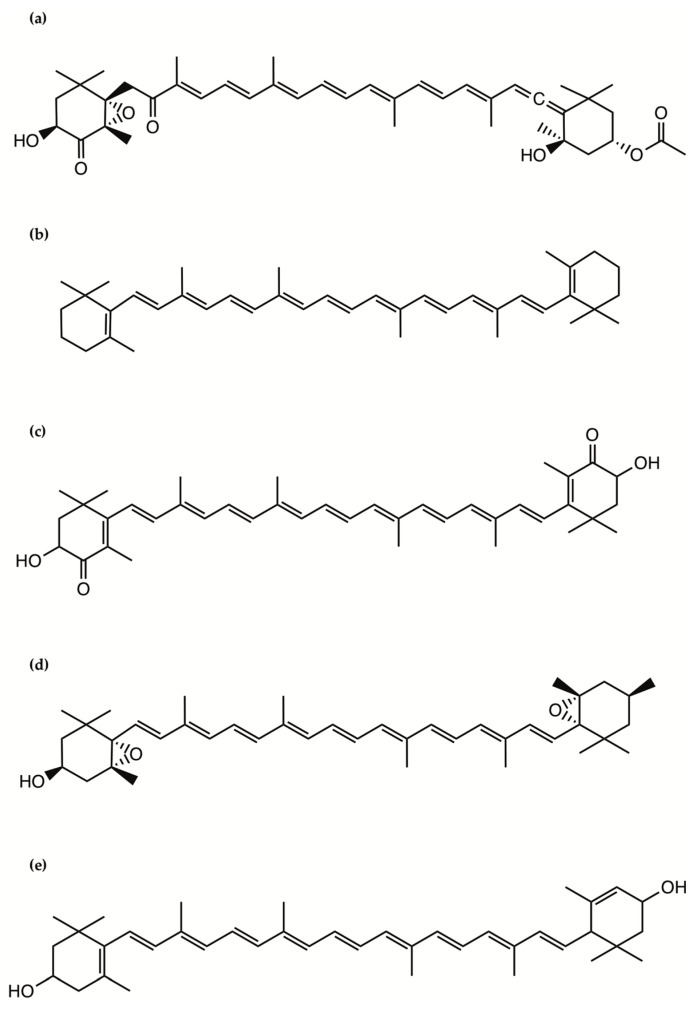
The chemical structure of the pigments: (**a**) Fucoxanthin; (**b**) β-carotene; (**c**) Astaxanthin; (**d**) Violaxanthin; (**e**) Lutein.

Fucoxanthin occurs abundantly in some macro- and microalgae and contributes to more than 10% of the estimated total production of carotenoids in nature. This pigment has been extensively investigated in microalgae for its role in photosynthesis. Fucoxanthin has been isolated and structurally identified from microalgae and can reach in a freeze-dried diatom a weight as high as 16.5 mg/g, which is 10 times higher than that in brown algae, suggesting potential applications in human and animal food, health and cosmetics [31]. Fucoxanthin is a xanthophyll, which contains an oxygen atom and thus is less chemically hydrophobic compared with the carotenes, which do not contain oxygen and are fat-soluble and insoluble in water (Figure 2). It includes a typical allenic bond, epoxide group, and conjugated carbonyl group in a polyene chain with antioxidant properties [31].

It has been found to have a number of therapeutic activities, including anti-obesity, anticancer, antioxidant, and anti-diabetic effects. In the last few years, nutrigenomics studies have focused on the exceptional ability of fucoxanthin in modulating the expression of specific genes involved in cell metabolism. Moreover, fucoxanthin improves the production of docosahexaenoic acid (DHA) [32]. Fucoxanthin exerts an anti-obesity activity by modulating the increase of reactive oxygen species (ROS) and the down-regulation of lipid metabolism genes. Fucoxanthin significantly reduces plasmatic and hepatic triglyceride concentrations and positively influences cholesterol-regulating enzymes such as 3-hydroxy-3-methylglutaryl coenzyme A reductase and acyl-coenzyme A [33]. In 2016, Jeong Hwa Kim et al. evaluated the anti-obesity effects of *Phaeodactylum tricornutum* powder based on a number of metabolic parameters in a model of diet-induced obesity (C57B/6 mice on a high–fat diet). They found that a range of 771.1 and 1273.18 μg/g are present in 15–30% of *P. tricornutum*. Fucoxanthin was micellized and transferred to the soluble fraction at the ileum in an in vitro simulated digestion system [34]. In particular, they observed that fucoxanthin restored adenosine monophosphate (AMP)-activated protein kinase (AMPK) phosphorylation and inhibited the activities of lipogenic enzymes such as acetyl-CoA carboxylase (ACC) and HMG-CoA reductase 3-hydroxy-3-methyl-glutaryl-coenzyme A reductase (HMGCR) in the livers of high fat diet-fed mice (Table 1). These findings provide an indication for new dietary anti-obesity therapies.

In their review, Martínez et al. [5] reviewed that fucoxanthin was reported by different authors to have anti-proliferative activity. Kotake-Nara et al. [35] found that fucoxanthin was one of the most active anti-cancer compounds among 15 types of carotenoids examined on three different prostate cancer cell lines (PC-3, Du145 and LNCaP). The percentage of viable cells after 72 h when fucoxanthin was added at 20 μM was 14.9% for PC-3, 5% for DU145 and 9.8% for LNCaP, respectively (determined by (3-(4,5-dimethylthiazol-2-yl)-2,5-diphenyltetrazolium bromide or MTT assay, for further details on this bioassay see Kotake-Nara et al., 2001). Recently, Neumann et al. [36] confirmed the antiproliferative effects of fucoxanthin extracted from *Phaeodactylum tricornutum*. The authors showed that fucoxanthin was able to reduce the metabolic activity of hepatocellular carcinoma (HepG2), adenocarcinoma of cervix (HeLa) and colonrectal adenocarcinoma (Caco-2) cells in a dose dependent manner (0.1, 1, 10 and 50 μg/mL). An inhibitory effect of up to 58% was measured in HepG2 cells. In HeLa and Caco-2 cells, the effect was stronger than that of the positive control with a final concentration of 5% dimethyl sulfoxide (DMSO). The authors also demonstrated that fucoxanthin increased caspase 3/7 activity up to 4.6-fold (Table 1).

Several studies have reported an effective radical scavenging ability of fucoxanthin. For example, Neumann et al. in 2019 [36] demonstrated antioxidant effects of fucoxanthin extracted from *Phaeodactylum tricornutum* on HeLa cells by using a 2,2-diphenyl-1-picryl-hydrazyl-hydrate assay (DPPH, for further details on this bioassay see Neumann et al., 2019). They observed that fucoxanthin had an IC_50_ value (measure indicating how much of a compound is necessary to inhibit cell proliferation by 50% in vitro) of 201.2 ± 21.4 μg/mL, while the value for ascorbic acid was 70.3 ± 18.7 μg/mL and for astaxanthin 79.32 ± 18.10 μg/mL. Moreover, the authors demonstrated that a ferric antioxidant power FRAP assay (for further details on this bioassay see Neumann et al., 2019) showed that fucoxanthin is equivalent to 64.74 ± 3.93 mmol Fe^2+^ per gram/dm, β-carotene to 6.55 ± 0.33 per gram/dm and astaxanthin to 63.97 ± 6.79 mmol Fe^2+^ per gram/dm. Finally, fucoxanthin was able to inhibit the oxidative burst in human progressive multifocal leukoencephalopathy (PML) cells, scavenge radicals and increase the glutathione/oxidized glutathione ratio (GSH/GSSG) (Table 1). Murakami et al. [37] screened 19 natural carotenoids for their structure-function relationship with respect to their radical scavenging activity. They found that the presence of an allenic bond, as seen in fucoxanthin increases the ability to inhibit the formation of superoxide in human promyelocytic HL-60 cells and of nitric oxide (NO) in mouse macrophage RAW 264.7 cells. Fucoxanthin significantly reduced reactive oxygen species (ROS) production and the viability of oxidatively-damaged monkey kidney fibroblast cells [38], human HaCaT keratinocytes [39], human hematoma HepG2 cells [40], and normal human hepatic L02 cells [41]. The antioxidant effect of fucoxanthin has also been reported in vivo. When oxidative stress was induced by a retinol deficiency in rats, fucoxanthin significantly reduced the lipid hydroperoxide levels of the plasma, liver, and liver microsomes [42]. Song Xia et al. [43] characterized the production and the activity of fucoxanthin isolated from the marine diatom *Odontella aurita*, demonstrating that fucoxanthin exhibited strong antioxidant properties, with an effective concentration for a 50% scavenging (EC_50_) of 1,1-dihpenyl-2-picrylhydrazyl (DPPH) radical and 2,2′-Azino-bis (3-ethylbenzthiazoline-6-6sulfonic acid (ABTS) radical of 0.14 and 0.03 mg/mL, respectively. Therefore, the results of this work suggested that *Odontella aurita* could be a natural source of fucoxanthin for human health and nutrition applications. Hosokawa et al. [44] demonstrated that fucoxanthin attenuated hyperglycemia in KK-Ay mice, but did not affect blood glucose levels in lean C57BL/6J mice. However, high-fat feeding could prompt obesity, hyperinsulinemia, high blood glucose, insulin resistance, and non-alcoholic fatty liver disease in C57BL/6J mice [45]. Maeda et al. [46] and Park et al. [45] showed that fucoxanthin significantly lowered the fasting blood glucose concentration, plasma insulin level, and insulin resistance index in diet induced obese mice. Fucoxanthin might reverse alterations in the lipid metabolism and insulin resistance induced by a high fat diet, at least in part, through reducing visceral fat mass, hyperinsulinemia, hepatic glucose production, and hepatic lipogenesis, and altering hepatic glucose-regulating enzyme activities [45]. Recently, Kawee-ai et al. [47] demonstrated that fucoxanthin isolated from *Phaeodactylum tricornutum* might also be useful for the prevention of obesity or diabetes by inhibiting carbohydrate-hydrolyzing enzymes and lipid accumulation and could be used as an ingredient for a functional food or dietary supplement (Table 1).

#### 2.1.2. β-Carotene

β-carotene is one of the typical primary carotenoids and is a component of the photosynthetic apparatus, which makes it necessary for photosynthesis. Microalgal-derived β-carotene has been reported to be more biologically active than synthetically produced β-carotene and can be considered as a “natural” food additive [48]. The microalga *Dunaliella salina* contains the highest amount of β-carotene (up to 10% of dry weight) compared to other algae in a closed tubular photobioreactor setting [49] with *Isochrysis* sp. containing the second highest amount [50]. *Dunaliella salina* is already commercially produced as a source of β-carotene [51] for use as an additive in food and feed applications, as well as for use in cosmetics and food supplements [52]. β-carotene is the most prominent member of the group of carotenoids that are a major class of fat-soluble pigments and antioxidants, and the intake of some carotenoids is associated with a reduced risk of disease through their involvement in cell signaling pathways. β-carotene, due to its antioxidant activity and its nutritional value as pro-vitamin A [53], has been widely applied in relation to food products and cosmetics.

β-carotene is a tetraterpenoid, consisting of 40 carbon atoms in a core structure of conjugated double bonds substituted with 2 β-ionone rings (Figure 2). Due to its extended system of 9 fully conjugated double bonds, β-carotene shows a major absorption peak in the visible spectrum with a maximum at 450 nm, responsible for the orange to red color of the compound. In biological systems, the predominant isomer is an all-trans β-carotene (E-isomer). However, cis-isomers have been found in living organisms and food samples [54], including 9-cis-, 13-cis-, and 15-cis- β-carotene (Z-isomers), in addition to several di- and poly-cis analogues [53].

β-carotene is used to ameliorate the secondary effects of the hereditary photosensitivity disorder erythropoietic protoporphyria, suggesting that carotenoids intercept the reaction sequence that leads to the formation of single oxygen. Singlet oxygen quenching by carotenoids occurs via physical or chemical quenching [55]. Physical quenching involves the transfer of the excitation energy form ^1^O_2_ to the carotenoid, resulting in a ground state oxygen and an excited triplet state carotenoid. In the process of physical quenching the carotenoid remains intact and can undergo further cycles of singlet oxygen quenching. β-carotene and other carotenoids (violaxanthin, zeaxanthin, astaxanthin) are the most efficient natural ^1^O_2-_quenchers. Their quenching activity is closely related to the number of conjugated double bonds present in the molecule [56]. β-carotene efficiently scavenge peroxyl radicals, especially at a low oxygen tension, and contributes to the defense against lipid peroxidation [57]. 

The antioxidant properties are related to the skin protective effects of β-carotene. It has been demonstrated that β-carotene levels in the skin and serum can be increased by supplementation with carotenoids derived from the alga *Dunaliella salina* [55]. In 2005, Murthy et al., conducted a research study using albino rats of either sex of the Wister strain, separated into five groups each of which maintained on the prescribed diet for a period of 15 days. The authors demonstrated a protective role for β-carotene rich algae in oxidative stress reduction (Table 1). Furthermore, β-carotene restores the activity of hepatic enzymes like catalase, peroxidase and superoxide dismutase, which in turn protects vital organs against xenobiotics and other damages. Treatments of rats with a toxin at 2 g/kg of body weight significantly reduced the level of catalase, peroxidase and superoxide dismutase by 84.88%, 118.11%, and 127.16%, respectively. However, pre-treatment of the rats with 250 μg/Kg and 125 μg/Kg of carotenoids preserved catalase, peroxidase and superoxide dismutase activities, findings which are comparable with the control values of the enzyme [58].

Epidemiological studies indicate that the incidence of cancer may be slightly lower among individuals with an above-average intake of β-carotene. Additionally, there is a correlation between β-carotene serum levels and a diminished risk of different kinds of cancer. Nishino et al. [59] completed two clinical trials where they demonstrated that β-carotene may be the most promising candidate as a cancer preventive agent. β-carotene was tested for its cancer-preventive activity in several interventional trials, e.g., two Linxian trials (Linxian 1 and Linxian 2), the Alpha-Tocopherol beta-Carotene (ATBC) Cancer Prevention Study, the β-carotene and Retinol Efficiency Trial (CARET), the Physicians’Health Study (PHS) and the Skin Cancer Prevention Study (SCPS) [60]. On the contrary, it has been shown in animal models that high doses of β-carotene affect the expression of a retinoic acid receptor subtype which might be important in the context of carcinogenesis [59]. These effects were more pronounced when the animals were additionally exposed to cigarette smoke [60]. In a study published by Singh et al., *Dunaliella salina* was grown under different stress conditions to enhance carotene production. The authors evaluated the cytotoxic activity of carotene on a breast cancer cell line (MCF-7), treated with 250 μg/mL for 72 h and observed an increase in cytotoxicity associated with carotene accumulation [61]. The contribution of β-carotene and other carotenoids to cancer prevention associated with a carotenoid-rich diet remains unclear (Table 1).

However, carotenoids, when used in association with the chemotherapy agent 5-fluorouracil facilitated a complete remission in colorectal cancer, rather than the partial remission as observed when chemotherapy was performed in the absence of additional metabolites [62].

#### 2.1.3. Astaxanthin

Astaxanthin, a carotenoid belonging to the xanthophyll class, has attracted great interest due to its antioxidant capacity and its possible role in reducing the risk of some diseases (Figure 2). Astaxanthin occurs naturally in microalgae such as *Haematococcus* sp., particularly the species *H. pluvialis* [63]. *Haematococcus* sp. is already commercially produced as a source of astaxanthin [63]. It is an important colorant in the salmonid and crustacean aquaculture feed industry, and in many countries it is also used as a dietary supplement. Its shares many of the metabolic and physiological activities attributed to carotenoids, including the presence of hydroxyl and carbonyl functional groups in the ketocarotenoids making them excellent antioxidants [64]. Astaxanthin is derived from β-carotene by 3-hydroxilation and 4-ketolation at both the ionone end groups. These reactions are catalyzed by β-carotene hydroxylase and β-carotene ketolase, respectively. Hydroxylation is widespread in higher plants, but ketolation is restricted to a few bacteria, fungi, and some unicellular green algae.

It can play a diversity of roles, e.g., in the prevention of some human pathologies, such as skin UV-mediated photo-oxidation, inflammatory processes, and even cancer [65].

Astaxanthin’s antioxidant capacities have been tested via in vitro lipid peroxidation and radical scavenging models as well as an in vivo vitamin E-deficient rat model [66]. Exposure to physiological stress, air pollution, tobacco smoke, chemicals or ultraviolet (UV) light, can improve the production of such agents. Oxidative damage has been linked to aging, atherogenesis, ischemia-reperfusion injury, infant retinopathy, age-related macular degeneration, and carcinogenesis. Dietary antioxidants, such as carotenoids, might help to prevent and fight several human diseases. Astaxanthin is very good at protecting membranous phospholipids and other lipids against peroxidation [66]. Palozza et al. demonstrated that the inhibitory effect of astaxanthin is comparable or superior to that of α-tocopherol in an egg yolk phosphatidylcholine liposomal suspension exposed to 2,2′-Azobis (2-amidinopropane) dihydrochloride (AAPH) [66]. Ranga Rao et al. [67] conducted a study to evaluate the bioavailability and antioxidant properties of carotenoids from a microalgal biomass tested in a rat model. A microalgal biomass containing 200 μM equivalent of β-carotene, astaxanthin and lutein per rat from *Haematococcus pluvialis* and *Botryococcus braunii* biomass, respectively, was dispersed in olive oil and administered to rats for a period of 15 days. The levels of these carotenoids in the plasma, liver and eye were examined by high performance liquid chromatography and also confirmed by mass spectroscopy. Astaxanthin accumulation in the group of rats fed with *H. pluvialis* was higher when compared to the *S. platensis* and *B. braunii* groups. The results indicate that astaxanthin from *H. pluvialis* has a better bioavailability and better antioxidant properties compared to other carotenoids [68]. In 2003, Spiller conducted an investigation by means of human safety study with a *H. pluvialis* algal extract with high levels of astanxanthin and confirmed that 6 mg of astaxanthin per day from an *H. pluvialis* algal extract can be safely consumed by a healthy adult (Table 1). These results indicate that astaxanthin is a more powerful antioxidant than other carotenoids [68]. Liu B.H. [69] and Bennedsen [70] conducted studies with Balb/cA mice. The authors investigated whether a dietary cell extract of *Haematococcus pluvialis* containing 2–3% astaxanthin could affect the bacterial load of *Helicobacter pylori* infected BALB/c A mice and whether it could induce a modulation of cytokine production. The BALB/c mice after two weeks of infection with *H. pylori* were orally fed with a cell extract of *H. pluvialis* (200 mg/kg body weight per day) for ten days. At the conclusions of the experiments, the authors observed a reduced bacterial load and gastric inflammation after treatment with an astaxanthin-rich algal meal. These effects were associated with a shift of the T-lymphocytes response from a predominant T helper type 1 (Th1) response dominated by Interferon gamma (IFN-γ) to a Th1/Th2 response with IFN-γ and Interleukin-4 (IL-4). A study conducted in 2008 [71] showed for the first time that orally administered total carotenoid and astaxanthin esters exert a dose dependent gastroprotective effect on acute, ethanol-induced gastric lesions in rats. Park et al. demonstrated that 8 mg of astaxanthin administered every day decreased one DNA damage biomarker while subjects fed with 2 mg astaxanthin also showed lower plasma C-reactive concentrations, demonstrating the anti-inflammatory action of astaxanthin in humans (Table 1). The immune markers significantly enhanced by means of feeding with astaxanthin included T cell and B cell mitogen-induced lymphocyte proliferation, NK cell cytotoxic activity, IFN-γ and Interleukin-6 (IL-6) production, and lymphocyte function-associated antigen 1 (LFA-1) expression [72].

In the literature there are numerous studies that report the importance of astaxanthin in relation to diabetes. Generally, patients with diabetes mellitus show very high oxidative stress levels, which are induced by hyperglycemia, due to dysfunction of pancreatic β-cells and tissue damage. Uchiyama et al. demonstrated that astaxanthin could reduce the oxidative stress caused by hyperglycemia in pancreatic β-cells and also improve glucose and serum insulin levels [73]. Astaxanthin can protect pancreatic β-cells against glucose toxicity (Table 1). It has also been shown to be an effective immunological agent in the recovery of lymphocyte dysfunctions associated with diabetic rats. Otton et al. demonstrated that astaxanthin could be a good adjuvant in prophylaxis or the recovery of lymphocyte dysfunctions associated with diabetic patients [74]. Additional studies have also shown that astaxanthin prevents diabetic nephropathy through the reduction of oxidative stress and renal cell damage [75]. Further, Mularczyk et al. [76] and Landon et al. [77] through their reviews highlighted the effects of an astaxanthin extract from *H. pluvialis* against the pathogenesis of diabetes and its chronic complications.

Astaxanthin has shown a significant anticancer activity when compared to other carotenoids like canthaxanthin and β-carotene. Very recently, Faraone et al. summarized in a review that astaxanthin can induce apoptosis through the down-regulation of anti-apoptotic protein (Bcl-2, p-Bad, and survivin) and the upregulation of proapoptotic (Bax/Bad and PARP) expression in neoplastic, colon, breast, prostate, and lung cells [78]. Palozza et al. demonstrated the growth-inhibitory effects of the astaxanthin-rich *H. pluvialis* on colon cancer cells (HCT116), decreasing the expression of *cyclin D1* and increasing *p53* and some cyclin kinase inhibitors, including *p21^waf-1/CIP-1^* and *p27*, which arrest cell cycle progression (Table 1). Moreover, it may also promote apoptosis through a down-regulation of the phosphorylation of protein kinase B (AKT), changes in the apoptosis-related proteins, including Bax, Bcl-2, and Bcl-Xl, and in mitogen-activated protein (MAP) kinase signalling [79].

#### 2.1.4. Violaxanthin

Violaxanthin is an orange-colored natural xanthophyll, a derivative of β-carotene which only differs for four peripheral groups: two-epoxy, at the positions 5, 6 and 5′, 6′and two hydroxy-, at the positions 3 and 3′. The polar groups are bonded to the β-ionone ring on two sides of each molecule (Figure 2).

**Figure 3:**
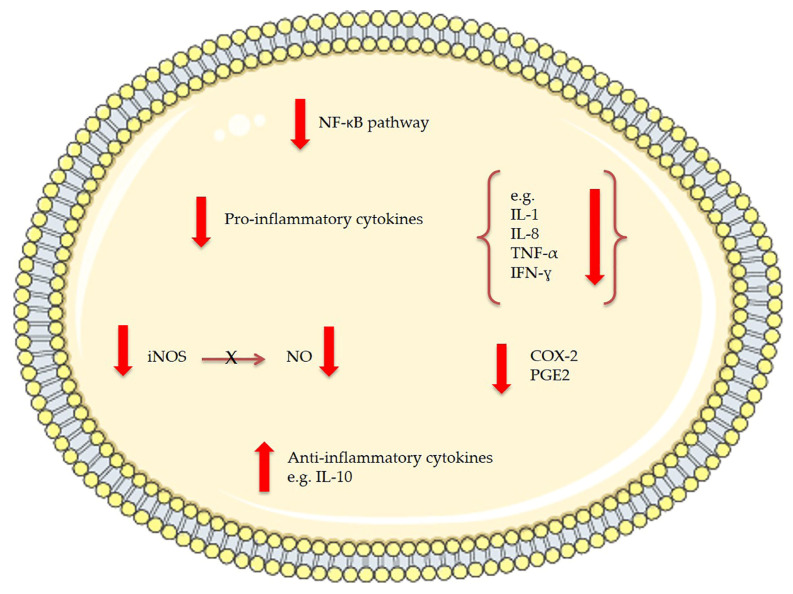
The effects of bioactive compounds extracted from microalgae involved in anti-inflammatory mechanisms. NF-kB stands for nuclear factor-kappa B, IL for interleukin, TNF for tumor necrosis factor, IFN for interferon, iNOS for inducible nitric oxide synthase, NO for nitric oxide, COX-2 for cyclo-oxygenase-2 and PGE2 for prostaglandin E2.

In 2011, Pasquet et al. [80] demonstrated that *Dunaliella tertiolecta* dichloromethane extract exhibited a strong anti-proliferative activity on human breast cancer cells (MCF-7) and human prostate cancer cells (LNCaP) but not on human lung carcinoma (A549) and human breast cancer cells (MDA-MB-231). Through high resolution mass spectrometry and spectrophotometric analysis violaxanthin was identified as the most anti-proliferative molecule present in the *Dunaliella tertiolecta* dichloromethane extract. Pasquet et al. showed that the sub-fraction containing violaxanthin inhibited MCF-7 growth (with 72 h exposure) at a concentration as low as 0.1 μg/mL and in a dose dependent manner from 0.1 μg/mL to 40 μg/mL (Table 1). However, despite indications of early apoptosis (phophatidylserine translocation detected using annexin-V-Alexa 568 fluorochrome), the violaxanthin sub-fraction did not cause any DNA fragmentation. Successively, Soontornchaiboon et al. [81] assessed the anti-inflammatory activity and mechanism of action of violaxanthin purified from *Chlorella ellipsoidea* using various assays, such as real-time polymerase chain reaction (RT-PCR), Western blotting and electrophoretic-mobility shift assay (EMSA). The anti-inflammatory effect of violaxanthin was demonstrated by the significant inhibition of nitric oxide (NO) and prostaglandin E_2_ (PGE_2_) (Figure 3). Violaxanthin effectively inhibited the LPS-mediated nuclear factor-κB (NF-κB) p65 subunit translocation into the nucleus, suggesting that violaxanthin anti-inflammatory activity may be based on the inhibition of the NF-κB pathway. The experiments showed that violaxanthin markedly inhibited NO production in LPS (1 μg/mL)-treated RAW 264.7 cells in a dose-dependent manner and this effect was maximal at 60 μM (Table 1).

#### 2.1.5. Lutein and Minor Carotenoids

Lutein is a yellow colored 40 carbon-long chain structured xanthophyll pigment [82] (Figure 2) and zeaxanthin is its stereoisomer, while neoxanthin has the characteristic structure of 5,6-monoepoxide and an allelic bond [83]. Lutein protects cells from ROS damage under stress conditions and, indeed, has attracted great attention due to its potential role in preventing or ameliorating age-related macula degeneration [82]. This antioxidant activity is thought to be responsible for reducing injury due to oxidative and inflammatory processes in cells and tissues. This carotenoid has also been proposed for the prevention of certain cancers [84] and for the protection of skin from UV-induced damage [85]. Lutein has been extensively used as a feed additive and a food coloration agent in industry [86]. Lutein together with neoxanthin and zeoxanthin have scavenging properties [87]. Knowledge of the biosynthetic pathways for lutein biosynthesis in microalgae is limited. It is now believed that all types of carotenoids, including lutein, are obtained from common five-carbon (C_5_) starting molecules isopentenyl diphosphate (IPP) and dimethylallyl diphosphate (DMAPP). These common metabolic precursors (IPP and DMAPP) might be derived from either one of two independent pathways: (1) the cytosolic mevalonate (MVA) pathway starting from Acetyl-CoA, or (2) the plastidic methylerythritol 5-phosphate (MEP) pathway starting from pyruvate [88]. There are evidences that the precursors for microalgal carotenoids including lutein biosynthesis proceed from the MEP pathway in *Dunaliella salina*, *Chlorella vulgaris*, *Scenedesmus* sp. [88], and *Haematococcus pluvialis* [89]. 

Cha et al. at the same time evaluated the anticancer activity of violaxanthin from *Chlorella ellipsoidea* [84] and lutein from *Chlorella vulgaris*, measuring their cytotoxicity and apoptosis-inducing activity. The authors showed that extracts of *Chlorella vulgaris* inhibited colon cancer (HCT116) cell growth in a dose-dependent manner, yielding IC_50_ values of 40.41 ± 4.43 μg/mL (Table 1). Kotake-Nara [83] demonstrated that neoxanthin reduces the viability of human prostate cancer cells inducing apoptosis in PC3 characterized by morphological changes, DNA fragmentation, an increased percentage of hypodiploid cells, and a cleaveage of caspase-3 and PARP. The viability of the cells significantly decreased after 72 h of incubation with 20 μM of neoxanthin, with the down-regulation of Bax and BCl-2 expression and a diminution in the levels of procaspase-3 and PARP.

These two natural compounds are involved in epidemiological and intervention trials that support a nutrient–health relationship in preventing age-related cataracts and maculopathy [90]. In fact, in the literature, both zeaxanthin and lutein are reported to play an important role in maintaining normal visual function [91]. Santocono [92] and co-workers investigated the antioxidant activity of lutein and zeaxanthin by using chemiluminescence techniques and found that these carotenoids have a similar superoxide-scavenging activity. Their investigation was conducted on SK.N.Sh human neuroblastoma and rat trachea epithelial cells subjected to oxidative stress by exposure to UVA radiation. Human neuroblastoma cells were irradiated with UVA for 30 min, 40 μM of carotenoids were added immediately after irradiation, and DNA repair was observed for 2 h. (Table 1). In the cell lines, irradiation with UVA resulted in time-dependent DNA damage. The effectivness of these carotenoids as antioxidants depends on a number of factors, but the addition of carotenoids after UVA exposure influences the kinetics of DNA repair in a very different manner.

**Table 1:** The table reports some of active pigments identified in microalgae. When available, mechanism of action, concentration used and inhibitory concentration values (IC_50_) are reported.

Compound	Microalgae	Bioactivity	Concentration	Mechanism of Action	Ref.
Fucoxanthin	*Phaeodactylm tricornutum*	Anti-obesity: (C57B/6 mice a high-fat diet).	In vivo: 771.1 and 1273.18 μg/g of diet for 15 and 30% PT powder.	Activation of AMPK and HMGCR pathways.	[34]
		Anticancer: (Caco-2, HeLa and HepG2).	In vitro: Dose–dependent manner (0.1, 1, 10 and 50 μg/mL).	Increased the caspase activity up to 4.6-fold.	[36]
		Antioxidant: (Human primary blood cells)	In vitro: IC_50_ value of 201.2 ± 21.4 μg/mL.	Inhibit the oxidative burst in human PMLs, scavenge radicals and increase the GSH to GSSH ratio.	[36]
		Anti-diabetic: (3T3-L1 cells)	In vitro: IC_50_ value of 0.68 mmol/L and 4.75 mmol/L	Inhibiting carbohydrate-hydrolyzing enzymes and lipid accumulation.	[47]
β-carotene	*Dunaliella salina*	Antioxidant: (Albino rats of either sex of the Wister strain weighing 180–220 gm)	In vivo: 125 μg/Kg and 250 μg/Kg.	Restores the activity of hepatic enzymes.	[58]
		Anticancer: (MCF-7 breast cancer)	In vitro: 250 μg/mL	Remains unclear.	[61]
Astaxanthin	*Haematococcus pluvialis*	Antioxidant: (Thirty-five healthy adults age 35–69 years)	In vivo: 6 mg/day	Remains unclear.	[68]
		Anti-inflammatory: (young healthy adult human female).	In vivo: 2 or 8 mg/daily.	Shifting the T-lymphocyte response from a *Th1* response dominated by IFN-γ to a Th1/Th2 response dominated by IFN- γ and IL-4.	[72]
		Anti-diabetic: (C57BL/KsJ-db/db mice).	In vivo: 10 mg/mouse/day.	Preservation of β cell function.	[73]
		Anticancer: (HCT116 colon cancer).	In vitro: 25 μg/mL.	Increase of *p53*, *p21^WAF-1/CIP-1^* and *p27* expression, decrease of *cyclin D1* expression and AKT phosphorylation.	[79]
Violaxanthin	*Dunaliella tertiolecta*	Anticancer: (MCF-7, LNcaP cell lines)	In vitro: From 0.1 μg/mL to 40 μg/mL.	Phophatidylserines translocation.	[80]
	*Chlorella ellipsoidea*	Anti-inflammatory: (Raw 264.7 cell lines).	In vitro: 60 μM	Inhibition of NF-κB	[81]
Lutein	*Chlorella vulgaris*	Anticancer: (HCT116 cell lines).	In vitro: IC_50_ values of 40.41 ± 4.43 μg/mL.	Apoptosis-inducing activity.	[84]
		Antioxidant: (human neuroblastoma cells, rat trachea epitelial cells).	In vitro: 40–50 μM.	Remain unclear	[92]

### 2.2. Polyphenols

Polyphenols are a group of compounds of about 8000 known molecules which are generally divided into ten different classes depending on their basic chemical structure [93] and are broadly divided in four classes: phenolic acids, flavonoids, stilbenes, and lignans [94]. Phenolic compounds are recognized as important natural antioxidants and extraction of polyphenols from natural resources has received enormous recent attention. Polyphenolic compounds isolated from marine algae exhibit a broad spectrum of beneficial biological properties including antioxidant, anticancer, anti-microbial, anti-inflammatory, anti-diabetic [95] and antiviral activities [96]. In this view, microalgal biomass exhibits great potential for target bioactive compound accumulation. Polyphenols act as antioxidants through single electron transfer and through hydrogen atom transfer [52]. Some studies suggest that the content of phenolic substances in microalgae is lower than or equal to the minimum amounts reported for terrestrial plants, and only include phenolic acids. The recent explosion of interest in the bioactivity of polyphenols is due to their potential health benefits as, for example, cardioprotective [97], anti-carcinogenic [98] and anti-diabetic [99] compounds. In a recent study [100], Del Mondo et al. investigated the structural variety and the beneficial activity of polyphenols, but they also highlighted the lack of genetic and biochemical information on their biosynthetic route in microalgae. Investigations on the polyphenol biosynthetic pathway in microalgae are required to further understand and thus exploit microalgal phenolic compounds.

Li et al. screened 23 microalgal species [101], Hajimahmoodi et al. (2010) screened another 12 species [102], Goiris et al. [52] screened 32 microalgae, and Safafar [87] screened six species for possible antioxidant capacity and correlated this activity with polyphenol content. These studies found that industrially-cultivated *Tetraselmis suecica*, *Isochrysis* sp., *Chlorella vulgaris*, and *Phaeodactylum tricornutum* possessed the highest antioxidant capacities and, thus, could be potential new sources of natural antioxidants. Recently, Patil L. examined the antioxidant activity of *Scenedesmus bajacalifornicus* BBKLP-07 [103] and they confirmed the presence of phenols using the Folin-Ciocalteu method (Table 2) of Singleton et al. [104]. The highest flavonoid content was observed in aqueous extracts. Flavonoids play a crucial role in protecting cells from premature aging and disease by shielding DNA, proteins and lipids from oxidative damage [105]. In addition, crude extracts also showed anti-diabetic, anti-inflammatory and anti-microbial activities. Anti-diabetic activity was demonstrated by the inhibition of α-amylase enzyme involved in the digestion of carbohydrates, the anti-inflammatory activity by the analysis of different molecular mediators (e.g., tumor necrosis factor TNF-α, interleukin 1, nitric oxide and prostaglandin) and antimicrobial activity was found against the foodborne pathogenic bacteria *Escherichia coli*, *Salmonella typhi*, *Bacillus subtilis*, and *Staphylococcus aureus* [103].

### 2.3. Polysaccharides

Polysaccharides are large molecules made by many smaller monosaccharides. Depending on which monosaccharides are connected, and which carbons in the monosaccharides connect, polysaccharides can have a variety of forms. Polysaccharides have been studied for a long time due to their characteristics, especially their chemical behaviour that is reflected by their conformation. Polysaccharides produced by microalgae have already proved to be promising agents in various fields, such as food, feed, pharmaceutical, and biomedical, due to their anti-viral, anti-bacterial, anti-oxidant, anti-inflammatory and immunomodulatory activity [106]. In microalgae, polysaccharide biosynthesis and polysaccharide sulfation take place through the Golgi apparatus (GA) [107]. Polysaccharide sulfonation in red microalgae was carried out by supplying *Porphyridium* cells with Na_2_^35^SO_4_ cysteine. Results suggested the role of cysteine as sulphur donor, with the intervention of the enzyme sulfotransferase that catalyzes the attachment of sulfur to cell-wall polysaccharides.

Sulphated polysaccharides (sPS) from marine microalgae, principally the ones produced by *Porphyridium*, have been reported to have anti-viral activity. The mechanism of action is not yet completely understood; the anionic nature of sPS makes them good candidates to protect against viruses. In 1996, Hayashi et al. [108] showed that sPS inhibited the penetration of viral particles into host cells (Table 2). In particular, they tested the inhibitory effects of calcium spirulan and dextran sulphate on the replication of Human immunodeficiency virus 1(HIV-1) and *Herpes simplex* virus (HSV-1) and demonstrated that the concentration of calcium spirulan and dextran sulphate required for 50% inhibition (IC_50_) was 9.3 and 9.6 μm/mL, respectively. 

Raposo et al. [109] showed the antiviral applications of exopolysaccharied (EPS) from marine microalgae, in particular against *Herpes simplex* and *Varicella zoster* viruses (HSVand VZV), human *cytomegaloviruses* (HCMV), measles, mumps and flu viruses, and vaccinia virus, a variola–related virus. In fact, the EPS from *Porphyridium purpureum* proved to be active against *Vaccinia* and *Ectromelia orthopoxvirus* infection. In studies conducted with HepG2 and VERO C1008 cells, IC_50_ was significantly lower (0.78 and 0.65 μg/mL respectively) than the response to dextran sulfate (1.24 μg/mL) [110]. In 2014 Raposo et al. tested the anti-microbial activity of the EPS from *Porphyridium cruentum* and reported that ethanolic extracts of this species showed some significant activity against *Salmonella enteritidis.* Tannin-Spitz [111] demonstrated that sulfated polysaccharides from *Porphyridium* exhibited antioxidant activity against the autoxidation of linoleic acid and inhibited oxidative damage to 3T3 cells that might be caused by FeSO_4_ (Table 2). The sulfated EPS from *Rhodella reticulata* also had antioxidant activity [112], with the crude polysaccharide being twice as strong as α-tocopherol. Polysaccharides from marine microalgae, like *Phaeodactylum tricornutum* and *Chlorella stimatophora*, had already been shown to have anti-inflammatory activity against paw edema induced by carrageenan. The anti-inflammatory efficacy was tested in vivo, by intraperitoneally injecting the crude polysaccharide in female rats and mice, and in vitro, by evaluating the phagocytic activity in macrophages from mice [113]. Guzman et al. also demonstrated the direct stimulatory effect of *P. tricornutum* on immune cells due to the positive phagocytic activity tested either in vitro or in vivo, and the immunosuppressant activity of sulfated polysaccharides from extracts of *Chlorella stigmatophora*. In 2007, Tabarsa et al. showed that the polysaccharides extracted from *Chlorella vulgaris* after fractionation appeared to stimulate macrophage cell lines (RAW264.7) via induction of NO, PGE_2_ and pro-inflammatory cytokine production with enhanced expression of their mRNA [114]. High molecular weight over-sulfated EPSs from *Porphyridium* inhibited neoplastic mammalian cell growth and the biomass of this marine microalgae was shown to prevent the proliferation of colon cancer in rats [115]. Gardeva et al. [116] showed that a sulfated polysaccharide derived from *Porphyridium cruentum* was active against Griffi myeloid tumor in hamsters both in vivo and in vitro (Table 2). When tested in vivo, this polysaccharide decreased transplantability in all experimental groups.

**Table 2:** The table reports some of active polyphenols and polysaccharides identified in microalgae. When available, mechanism of action, concentration used and inhibitory concentration values (IC_50_) are reported.

Compound	Microalgae	Bioactivity	Concentration	Mechanism of Action	Ref.
Polyphenols					
Flavonoids and alkaloid	*Scenedesmus bajacalifornicus BBKLP-07*	Antioxidant	In vitro: Radical scavenging effects of 60.45 and 63.57% at 50 μg/mL.	Reduction of methanolic solution of colored free radical DPPH by free radical scavengers.	[103]
		Anti-diabetic	In vitro: IC_50_ 80.21 μg/mL	Inhibitory activity of α-amylase.	[103]
		Anti-inflammatory	In vitro: 67.35% protein denaturation at 100 μg/mL		[103]
Polysaccharides					
Calcium spirulan and dextran sulphate	*Porphyridium cruentum*	Anti-viral	In vitro: IC_50_ 9.3 and 9.6 μm/mL.	Inhibitory effect on the replication of HIV-1 and HSV-1.	[108]
	*Porphyridium UTEX 637*	Antioxidant: (3T3 cells)	In vitro: 7.5 μg/well: 41.4% of inhibition. 19 μg/well: 65% of inhibition. 37.5 μg/well: 79.7% of inhibition.	Autooxidation of linoleic acid, and oxidative damage to 3T3.	[111]
	*Chlorella stigmatophora* and *Phaeodactylm tricornutum*	Anti-inflammatory: Female C57BI mice	In vivo: Intraperitoneally crude polysaccharide extract 5 or 10 mg/kg.	Colloidal carbon clearance (in vivo *assay)* Phagocytic activity (in vitro assay)	[113]
	*Porphyridium cruentum*	Anticancer: Golden Syrian race Graffi Myeloid tumor	In vitro: Dose dependent manner at different time.	Increased both, spreading and phagocytic activity of peritoneal macrophages in healthy and GTBH in a dose dependent manner.	[116]

### 2.4. Lipids

Microalgae are known to be excellent producers of valuable lipids, such as fatty acids, polar lipids, oxylipins, and steroids with possible applications as nutrient supplements, as well as in the pharmaceutical, cosmeceutical and biofuel sectors. Approximately 2400 tons of microalgae biomass are marketable per year for health applications and the market size of recommended omega-3 based pharmaceuticals alone represents 1.5 billion dollars [117]. Lipid content in microalgae can reach 25% of dry weight, but can be increased by applying different methodologies. Biological fatty acids are composed of a hydrocarbon chain with one terminal carboxyl group (COOH). 

Lipids are generally amphipathic (part of their structure is hydrophilic and another part is hydrophobic) and this property is the key for their role as fundamental components of cellular and organelle membranes, as well as their industrial applications [118]. Several studies have focused on implementing lipid production, via classical culturing parameter modifications or via metabolic engineering, especially for biofuel applications [119,120]. Lipid synthesis has been extensively studied [119], even if metabolic pathways are not completely characterized for all the microalgal classes. Various enzymes involved in lipid synthesis have been often considered for genetic engineering modifications in order to implement lipid production, especially for nutraceutical and biofuel applications [119]. Microalgal bioactivity screening and lipid activity evaluation identified different possible applications for prevention and treatment of various human pathologies: anticancer, antioxidant, anti-inflammatory, and others (Table 3). Regarding fatty acids, the two most important long-chain omega-3 (ω-3) polyunsaturated fatty acids (PUFAs), such as eicosapentaenoic acid (EPA) and docosahexaenoic acid (DHA), have been found to have possible beneficial activities for several pathologies, such as arteriosclerosis, hypertension, inflammation, cancer, rheumatoid arthritis, and asthma microbial and viral infections, as well as retinopathy and mental health [121,122,123,124,125,126]. For example, an EPA-enriched fraction from the diatom *Cocconeis scutellum* Ehrenberg (Bacillariophyceae) had antiproliferative activity on breast carcinoma (BT20) cells [127], with activation of caspase-3 and caspase-8, and cell cycle progression block from S to G2-M phases [127]. Desbois et al., 2008 [128] isolated from the diatom *Phaeodactylum tricornutum* the monounsaturated fatty acid (9Z)-hexadecenoic acid (palmitoleic acid; C16:1 n-7) and the relatively unusual polyunsaturated fatty acid (6Z, 9Z, 12Z)-hexadecatrienoic acid (HTA; C16:3 n-4) and tested them for antimicrobial bioactivity. They found that palmitoleic acid inhibited the growth of staphylococcal species, including multidrug-resistant *Staphylococcus aureus* (MRSA), and the growth of the food-borne pathogen, *Bacillus weihenstephanensis*. HTA inhibited the growth of Gram-positive and Gram-negative bacteria, such as *S. aureus*, *Staphylococcus epidermidis* and also two marine bacteria, *Planococcus citreus* and *Listonella anguillarum*. Inhibitory concentration (IC_50_) values were calculated for activities against *S. aureus* with values ranging from 10 to 20 and 20 to 40 μM for palmitoleic acid and HTA, respectively.

Gutiérrez-Pliego et al. [129] proposed microalgal n-3 fatty acids in substitution to fish oil for the treatment of diabetes and prevention of the appearance of health complications caused by inflammatory processes. They analysed the effects of supplementation with n-3 fatty acids (EPA and DHA) extracted from microalgae (Chlorophyceae and Eustigmatophyceae) on the inflammatory markers from two different strains of mice, db/db and CD1. They observed that this supplementation induced an increase of the cytokines IL17A, IL-12, IL-4, IL-6, IL-10, and TGF-β, but a decrease of IFN-γ, TNF-α, and IL-5 in diabetic mice.

**Table 3:** The table reports active lipids identified in microalgae. When available, mechanisms of action, concentrations used and inhibitory concentration values (IC_50_) are reported.

Compound	Microalgae	Bioactivity	Concentration	Ref.
*Fatty acids*				
Palmitoleic acid and hexadecatrienoic acid (HTA)	*Phaeodactylum tricornutum*	Antimicrobial: Palmitoleic acid inhibited the growth of staphylococcal species, including multidrug-resistant Staphylococcus aureus MRSA. HTA inhibited the growth of Gram-positive and Gram-negative	In vitro: IC_50_ values of palmitoleic acid and HTA against S. aureus were 10–20 and 20–40 μM, respectively	[128]
EPA-enriched fraction	*Cocconeis scutellum* Ehrenberg	Antiproliferative activity on breast carcinoma (BT20) cells, activation of caspases-3 and caspase-8, and cell cycle progression block from S to G2-M phases	In vitro: Tests at 0–1.7 and 0.1–4 μg/well	[127]
EPA and DHA	*Chlorophyceae* and *Eustigmatophyceae*, species names not specified	Antidiabetes: increase of the cytokines IL17A, IL-12, IL-4, IL-6, IL-10, and TGF-β but the decrease of IFN-ɣ, TNF-α, and IL-5 in diabetic mice	In vivo: 1 mg/g of mouse weight.	[129]
*Polar lipids*				
Two monogalactosyldiacyl glycerolipids (MGDGs	*Phaeodactylum tricornutum*	Pro-apoptotic activity on immortal mouse epithelial cell lines (W2 cells).	In vitro: 52 μM and 64 μM	[130]
Two MGDGs	*Tetraselmis chuii*	Anti-inflammatory: reduce nitric oxide (NO) production and inducible nitric oxide synthase (iNOS) protein levels in lipopolysaccharide (LPS)-stimulated RAW264.7 macrophage cells	In vitro: 50 μg/mL	[131]
MGDGs and digalactosyl diacylglycerolipids (DGDGs)	*Nannochloropsis granulata*	Anti-inflammatory: reduce nitric oxide (NO) production and inducible nitric oxide synthase (iNOS) protein levels in lipopolysaccharide (LPS)-stimulated RAW264.7 macrophage cells	In vitro: 50 μg/mL	[132]
sulfoquinovosyl diacylglycerolipids (SQDGs)	*Tetradesmus lagerheimii*, *Scenedesmus producto-capitatus*, *Pectinodesmus pectinatus*, *Tetradesmus wisconsinensis*	inhibit the glutaminyl cyclase (QC)	In vitro: 0.2 mg/mL	[133]
A synthetic sulfolipid (Sulfavant) SQDG18	*Thalassiosira weissflogii CCMP1336*	It triggered an effective immune response against cancer cells to improve dendritic cell (DC) maturation and increase CD83-positive DC. SQDG18 stimulated the production of the pro-inflammatory cytokines IL-12 and INF-ɣ		[134,135]
MGDGs DGDGs	*Chlorella vulgaris*	Antitumor: Epstein-Barr virus-associated early antigen (EBV-EA) activation on Raji cells induced by 12-O-tetradecanoylphorbol-13- acetate (TPA)	In vitro: 500–2500 mol ratio/TPA	[136]
SQDG	*Porphyridium cruentum*	Inhibition of the growth cancer cell-lines on human colon (DLD-1), breast (MCF-7), prostate adenocarcinoma (PC-3) and malignant melanoma (M4 Beu) cancer cells;	In vitro: IC_50_: 20–46 µg/mL	[137]
SQDG	*Porphyridium cruentum*	Inhibition of DNA α-polymerase;	In vitro: IC_50_: 378 µg/mL	[137]
Lipid extracts containing EPA, SQDG, MGDG, DGDG and others	*Pavlova lutheri*	Down-regulation of the production of cytokine IL-6 in lipopolysaccharide (LPS)-stimulated human THP-1 macrophages; Down-regulation of Toll-like receptor 8, Toll-like receptor 1, TNF receptor-associated factor 5, Mitogen-activated protein kinase 1; Increase of Prostaglandin E receptor 1	In vitro: 3 µg/mL total fatty acids	[138]
Oil containing eicosapentaenoic acid (EPA), phospholipids and glycolipids	*Nannochloropsis oculata*	Glycolipids in the algal oil may increase Long-chain omega-3 polyunsaturated fatty acids (LC n-3 PUFA) bioavailability	In vivo: 5 mL algal oil per kg body weight each day per 7 days in rats	[139]
Oxilipins				
PUAs (2-trans,4-trans-decadienal, 2-trans,4-trans-octadienal and 2-trans,4-trans-heptadienal)	Pure compounds from Sigma-Aldrich Inc.	Anticancer (COLO 205 and A549 cells)	In vitro: 2–10 µM	[140]
2-trans-4-cis-7-cis-decatrienal, 2-trans-4-trans-7-cis-decatrienal and 2-trans-4-trans-decadienal	*Thalassiosira rotula*, *Skeletonema costatum* and *Pseudonitzschia delicatissima*	Anticancer (Caco-2 cells)	In vitro: 11–17 µg/mL	[141]
Oxylipin-containing lyophilised (OLM) biomass	*Chlamydomonas debaryana*	Anti-inflammatory activities on a recurrent 2,4,6-trinitrobenzenesulfonic acid (TNBS)-induced colitis mice model; significant decrease of TNF-α, iNOS and COX-2	In vivo: 300 and 600 mg/kg	[142]
Oxylipin 13-HOTE	*Chlamydomonas debaryana*,	Anticancer: UACC-62 (melanoma) than towards HT-29 (colon adenocarcinoma) cells	In vitro: 68.2 ± 0.2 µM (UACC-62) >100 µM (HT29)	[142]
15-HEPE	*Nannochloropsis gaditana*	Anticancer: UACC-62 (melanoma) than towards HT-29 (colon adenocarcinoma) cells	In vitro: 78.8 ± 4.6 µM (UACC-62) >100 µM (HT29)	[142]
Steroids				
Ergosterol, 7-Dehydroporiferasterol, Ergosterol peroxide, 7-Dehydroporiferasterol peroxide, 7-oxocholesterol	*Chlorella vulgaris*	Anti-inflammatory (12-O-tetradecanoylphorbol-13-acetate (TPA)-induced inflammation in mice)	In vivo: 0.2–0.7 mg/ear	[143]
Ergosterol peroxide	*Chlorella vulgaris*	Anticancer (TPA tumor-promoting effect in 7,12-dimethylbenz[a]anthracene-initiated mice)	In vivo: 2 μmol	[143]
Ergosterol, 7-Dehydroporiferasterol, mixture	*Dunaliella tertiolecta*	Anti-inflammatory activity on peripheral blood mononuclear cells (PBMC; isolated from sheep) treated with Concanavalin A (Con A) and lipopolysaccharide (LPS); increase of the anti-inflammatory cytokine interleukin 10 (IL-10)	In vitro: 0.4 mg/mL mixture; 0.8 mg/mL for ergosterol alone	[144]
Ergosterol, 7-Dehydroporiferasterol	*Dunaliella tertiolecta*	Neuromodulatory action was found in selective brain areas of rats	In vivo: 20–30 mg/kg	[145]
24-Oxocholesterol acetate, Ergost-5-en-3β-ol, Cholest-5-en-24-1,3-(acetyloxy)-, 3β-ol and others	*Isochrysis galbana*	Antituberculosis	In vitro: Minimum inhibitory concentration of 50–60 μg/mL	[146]
Stigmasterol, 5β-Hydroxysitostanol	*Navicula incerta*	Anti-cancer in human hepatoma HepG2 cells	40%, 43% and 54% toxicity at 5, 10 and 20 μM, respectively	[147,148]

### 2.5. Glycolipids

The principal characteristic of a glycolipid is the presence of a monosaccharide or oligosaccharide bound to a lipid moiety. The most common lipids in cellular membranes are the glycerolipids and sphingolipids, which have glycerol or a sphingosine backbone, respectively [149]. Glycolipids are located in the membrane of chloroplasts and thylakoids, and are considered important signal and regulatory molecules [150,151]. They are mainly composed by three classes including monogalactosyl diacylglycerols (MGDGs), digalactosyl diacylglycerols (DGDGs) and sulfoquinovosyl diacylglycerols (SQDGs). MGDGs present both anti-inflammatory and anti-cancer activities while SQDGs present immunostimulatory activities and inhibit the enzyme glutaminyl cyclase, which is involved in Alzheimer’s disease. 

Andrianasolo et al. [130] found two MGDGs in extracts of the diatom *Phaeodactylum tricornutum* which showed in vitro pro-apoptotic activity on immortal mouse epithelial cell lines (W2 cells). Two MGDGs from the microalga (Chlorophyta) *Tetraselmis chuii* and other MGDGs and DGDGs from *Nannochloropsis granulata* (Ochrophyta, Eustigmatophyceae) were able to reduce nitric oxide (NO) production and inducible nitric oxide synthase (iNOS) protein levels in lipopolysaccharide (LPS)-stimulated RAW264.7 macrophage cells [131,132] showing anti-inflammatory properties. SQDGs also showed interesting properties. In particular, sulfolipids extracted from the green microalgae (Chlorophyta) *Tetradesmus lagerheimii* (formerly *Scenedesmus acuminatus*), *Scenedesmus producto-capitatus*, *Pectinodesmus pectinatus* (formerly *Scenedesmus pectinatus*), and *Tetradesmus wisconsinensis* were able to inhibit glutaminyl cyclase (QC) [133], an enzyme involved in Alzheimer’s disease progression [152] and were suggested as possible lead compounds against Alzheimer’s disease. A synthetic sulfolipid derived from *Thalassiosira weissflogii* CCMP1336 (Bacillariophyta), named SQDG18, was able to trigger an effective immune response against cancer cells to improve dendritic cell (DC) maturation and increase CD83-positive DC. In addition, SQDG18 (Sulfavant) stimulated the production of the pro-inflammatory cytokines IL-12 and INF-γ and was suggested as a potent vaccine adjuvant [134,135]. SQDG18 and its derivatives were patented for possible use as vaccine adjuvants (EP3007725 A1; WO2014199297A1), as they are suitable for co-administration with antigens in vaccines for bacterial and viral diseases [153]. 

Crude sulfoglycolipidic fraction from *Porphyridium cruentum* showed antiproliferative activity on human colon (DLD-1), breast (MCF-7), prostate adenocarcinoma (PC-3) and malignant melanoma (M4 Beu) cancer cells (20–46 µg/mL), inhibition of DNA α-polymerase (IC_50_: 378 µg/mL), and inhibition of superoxide generation by activated peritoneal mono nuclear cells (IC_50_: 29.5 µg/mL) [137]. Lipid extracts, including EPA, SQDG, MGDG, and DGDG, from *Pavlova lutheri* induced the down-regulation of cytokine IL-6 in lipopolysaccharide (LPS)-stimulated human THP-1 macrophages, Toll-like receptor 8, Toll-like receptor 1, TNF receptor-associated factor 5, Mitogen-activated protein kinase 1, and the increase of Prostaglandin E receptor 1 [138]. MGDG synthase (MGD), UDP-sulfoquinovose synthase (SQD1), and sulfoquinovosyltransferase (SQD2) sequences are the enzymes which are suggested to be involved in MGDG and SQDG synthesis and have been identified in several microalgal species [8,14]. 

### 2.6. Steroids

Steroids are all composed by 17 carbon atoms arranged in four rings conventionally denoted by the letters A, B, C, and D-bonded to 28 hydrogen atoms [154]. Phytosterols have been used as additives in many food products, such as spread, dairy products, and salad dressing, and have received great attention because they are known to reduce cholesterol concentration of blood and prevent cardiovascular disorders [155]. 

*Isochrysis galbana*, *Nannochloropis gaditana*, *Nannochloropsis* sp. and *Phaeodactylum tricornutum* have phytosterol content ranging from 7 to 34 g per kg [156]; *Pavlova lutheri*, *Tetraselmis* sp. *M8* and *Nannochloropsis* sp. *BR2* may have phytosterol ranging from 0.4–2.6% dry weight, while 5.1% dry weight of phytosterol could be achieved for *P. lutheri* [157]. In particular, ergosterol, 7-dehydroporiferasterol, ergosterol peroxide, 7-dehydroporiferasterol peroxide, and 7-oxocholesterol from *Chlorella vulgaris* had anti-inflammatory activity in 12-O-tetradecanoylphorbol-13-acetate (TPA)-induced inflammation model in mice (EC50 0.2–0.7 mg/ear), with ergosterol peroxide inducing 77% reduction in tumour progression at 2 μmol [143]. A mixture of sterols and single sterols from *Dunaliella tertiolecta* were tested on peripheral blood mononuclear cells (PBMC; isolated from sheep) treated with Concanavalin A (Con A) and lipopolysaccharide (LPS), and anti-inflammatory capacity and induction of cytokins were evaluated. The mixture of ergosterol and 7-dehydroporiferasterol showed a suppressive effect on cell proliferation, reduction of pro-inflammatory cytokines production and the increase of the anti-inflammatory cytokine interleukin 10 (IL-10) [144]. Ergosterol and 7-dehydroporiferasterol from *D. tertiolecta* orally administered in rats also showed neuromodulatory activity in selective brain areas [145]. Finally, sterols from *Isochrysis galbana* showed anti-tuberculosis activity (Minimum inhibitory concentration of 50–60 μg/mL against *M. tuberculosis*; [146]), while sterols (in particular, Stigmasterol and 5β-Hydroxysitostanol) from *Navicula incerta* showed 40%, 43%, and 54% toxicity at 5, 10, and 20 μM, respectively, in human hepatoma HepG2 cells [147].

### 2.7. Oxylipins

**Figure 4:**
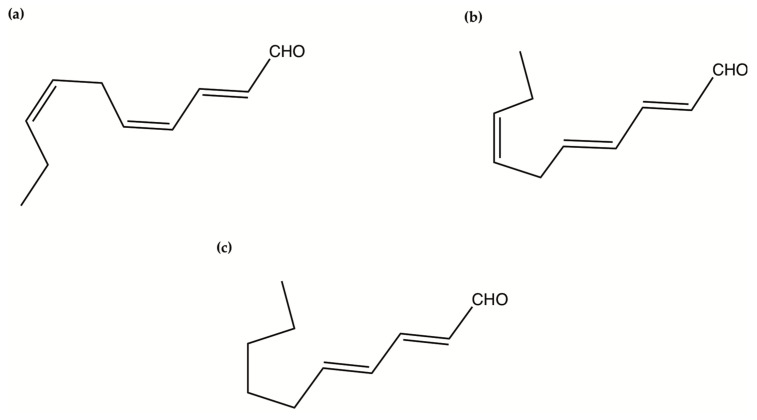
The chemical structure of polyunsaturated aldehydes. (**a**) 2-trans-4-cis-7-cis-decatrienal; (**b**) 2-trans-4-trans-7-cis-decatrienal; (**c**) 2-trans-4-trans-decadienal.

Oxylipins derive from the oxidation of polyunsaturated fatty acids and include polyunsaturated aldehydes (PUAs), known as volatile oxylipins, and non-volatile compounds which are other fatty acid derivatives with hydroxy-, keto-, oxo-, and hydroxy-epoxy units. In particular, Miralto and co-workers [141] isolated for the first time three PUAs (i.e., 2-trans-4-cis-7-cis-decatrienal, 2-trans-4-trans-7-cis-decatrienal and 2-trans-4-trans-decadienal) from the marine diatoms *Thalassiosira rotula*, *Skeletonema costatum* and *Pseudonitzschia delicatissima* (Figure 4). After their discovery, several other oxylipins were identified [158,159] with several studies on their ecological role and effects on predators (e.g., antipredator, allelopathic, antimicrobial activities) [160,161,162,163,164,165,166,167,168,169,170,171,172,173,174], along with possible biotechnological applications [140,141]. In particular, Miralto et al. showed that 2-trans-4-cis-7-cis-decatrienal, 2-trans-4-trans-7-cis-decatrienal and 2-trans-4-trans-decadiena had anti-proliferative activity on colon carcinoma Caco-2 cells at 11–17 µg/mL and found apoptosis induction by terminal deoxynucleotidyl transferase dUTP nick end labeling (TUNEL) assay. Successively, Sansone et al. (2014) tested 2-trans,4-trans-decadienal (DD), 2-trans,4-trans-octadienal (OD) and 2-trans,4-trans-heptadienal (HD) on the adenocarcinoma cell lines lung A549 and colon COLO 205, and the normal lung/brunch epithelial BEAS-2B cell line. DD was the strongest, while OD was the least active of the three PUAs. The activated death signaling pathway was evaluated in A549, for which the activity was stronger, and showed that cells treated with DD activated Tumor Necrosis Factor Receptor 1 (TNFR1) and Fas Associated Death Domain (FADD) by leading to necroptosis via caspase-3 without activating the survival pathway Receptor-Interacting Protein (RIP). HD activated the Fas/FADD/caspase pathway, while OD activated the TNFR1/FADD/caspase pathway and also RIP. An oxylipin-containing lyophilised (OLM) biomass, where the major oxylipin constituent was (9Z,11E,13S,15Z)-13-hydroxyoctadeca-9,11,15-trienoic acid ((13S)-HOTE), from *Chlamydomonas debaryana* had anti-inflammatory activities on a recurrent 2,4,6-trinitrobenzenesulfonic acid (TNBS)-induced colitis mice model [175]. In particular, OLM induced a significant decrease of pro-inflammatory cytokines (e.g., tumor necrosis factor TNF-α), cyclo-oxygenase-2 COX-2 and inducible nitric oxide synthase iNOS [175]. Oxylipins isolated from the microalgae *Chlamydomonas debaryana* (13-HOTE) and *Nannochloropsis gaditana* (15-HEPE) had antiproliferative activity against UACC-62 (melanoma) and HT-29 (colon adenocarcinoma) cells [142]. The oxylipins reduced ATP levels of both cell lines, suggesting a possible link with the cytotoxicity. Finally, 13-HOTE was combined with the anticancer drug 5-fluorouracil, inducing a synergistic activity on HT-29 cells. 

### 2.8. Proteins and Peptides

Various studies have shown that microalgal proteins/peptides can have different bioactivities (e.g., antioxidant, anticancer, antihypertensive, anti-atherosclerotic, anti-UV radiation and anti-osteoporosis [10,176]; Table 4). Few microalgal peptides have entered the clinical phase and even fewer have reached the market. A successful example is Dermochlorella^®^, an oligopeptide purified from the microalgae *Chlorella vulgaris*, which helps to firm the skin, reduces the colour of the stretch marks, increases expression of collagen, elastin, laminin and elafin, and restores skin elasticity [177]. Most of the peptides from microalgae have been obtained by enzymatic hydrolysis treatment (e.g., using alcalase, α-chymotrypsin, neutrase, papain, pepsin, pronase-E, and trypsin). 

Antioxidant activities have been found for the peptide VECYGPNRPQF from the green algae *Chlorella vulgaris* [178], which also exhibited gastrointestinal enzyme resistance and did not show cytotoxicity in human lung fibroblast WI-38 cell lines. Antioxidant activities have also been reported for the peptide LNGDVW from the green alga *C. ellipsoidea* [179], and two peptides, NIPP-1 (PGWNQWFL) and NIPP-2 (VEVLPPAEL), from for the benthic diatom *Navicula incerta* [180]. Regarding anticancer peptides, Sheih et al. [181] isolated the peptide VECYGPNRPQF from *C. vulgaris* with strong antiproliferative activity (inhibitory concentration value or IC_50_ 70.7 ± 1.2 μg/mL; post-G1 cell cycle arrest) in gastric cancer AGS cells without cytotoxicity in normal lung fibroblast WI-38 cells. The polypeptide CPAP from *Chlorella pyrenoidosa* showed antiproliferative activity on human liver cancer HepG2 cells (IC_50_ 426 μg/mL). In addition, experiments of CPAP micro- and nanoencapsulation demonstrated the resistance of CPAP to gastrointestinal enzymatic degradation [182] Antihypertensive activity (by the inhibition of the angiotensin I-converting enzyme ACE in the renin-angiotensin aldosterone system) was observed for the peptide VECYGPNRPQF from *C. vulgaris* [178] and two peptides, GMNNLTP and LEQ from the flagellate *Nannochloropsis oculata* [183]. A *Chlorella* derived peptide also showed anti-UV effects on skin fibroblasts after UVB irradiation by diminishing UVB-induced matrix metalloproteinases *MMP-1* and cysteine-rich 61 *CYR61* mRNA expression [184] and, hence, was suggested as UV protectant and anti-photoaging. Finally, the peptide MPDW isolated from *Nannochloropsis oculata* showed interesting anti-osteoporosis activity by promoting osteoblast differentiation, increasing expression of several osteoblast phenotype markers (e.g., alkaline phosphatase *ALP*, osteocalcin, collagen type I, *BMP-2*, *BMP2/4*) and bone mineralization in both human osteoblastic cells (MG-63) and murine mesenchymal stem cells (D1) [185]. 

**Table 4:** The table reports active peptides identified from microalgae, known mechanism of action and references.

Compound	Microalgae	Mechanism of Action	Ref.
VECYGPNRPQF	*Chlorella vulgaris*	Superoxide radical quenching	[178]
LNGDVW	*Chlorella ellipsoidea*	Free radical scavenging	[179]
PGWNQWFL, VEVLPPAEL	*Navicula incerta*	Cytotoxicity in HepG2/CYP2E1 cells	[180]
VECYGPNRPQF	*Chlorella vulgaris*	AGS cells	[181]
polypeptide CPAP	*Chlorella pyrenoidosa*	HepG2 cells	[182]
VECYGPNRPQF	*Chlorella vulgaris*	ACE inhibitor	[178]
GMNNLTP; LEQ	*Nannochloropsis oculata*	ACE inhibitor	[183]
VECYGPNRPQF	*Chlorella sp*	Gene expression inhibition of vascular adhesion molecules (E-selectin, ICAM, VCAM, MCP-1 and ET-1)	[186]
*Chlorella* derived peptide	*Chlorella* sp.	Inhibition of *MMP-1*, *CYR61*	[184]
MPDW	*Nannochloropsis oculata*	Increase of ALP, osteocalcin, collagen type I, BMP-2, BMP2/4; phosphorylation of MAPK/Smad pathways	[185]

**Figure 5:**
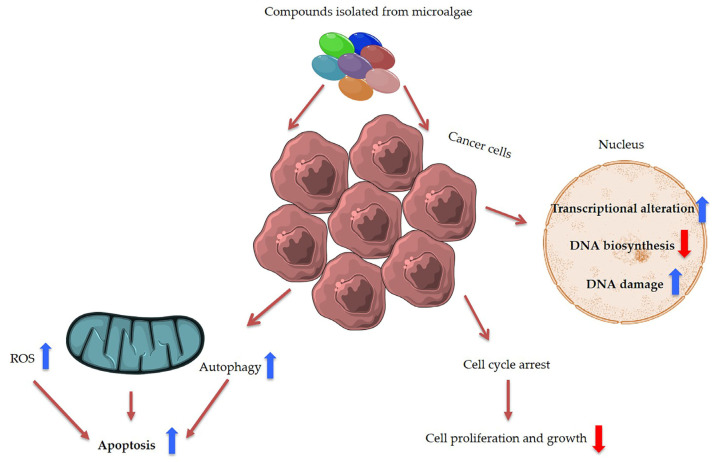
Summary of main anti-cancer effects induced by microalgal compounds. ROS is the abbreviation for reactive oxygen species.

Microalgal glycoproteins have also been reported, especially from *Chlorella vulgaris*, with immunostimulant activity in vitro and in vivo [187]. Tanaka et al. (1998) found that *Chlorella* glycoprotein (glycoprotein extract named CVS) induced antitumor effects (MethA and MethI fibrosarcomas of BALB/c origin and EL-4 lymphoma of C57BL/6 origin were used) against both spontaneous and experimentally induced metastasis in mice. They observed antimetastatic immunity through T cell activation in lymphoid organs and found that *Chlorella* glucoprotein enhanced the recruitment of these cells to the tumor sites. A glycoprotein, named ARS2 and with the sequence VGEAFPTVVDALVA, was purified from *Chlorella vulgaris* with antitumor activity on methylcholanthrene induced Meth A fibrosarcoma cells of BALB/c origin [188]. Successively, Hasegawa et al. (2002) suggested the involvement of the Toll-like receptor 2 in ARS2 antitumor activity [189] (Figure 5).

Microalgae have been shown to also produce particular peptides, such as taurine (2-aminoethanesulfonic acid), first discovered in the bile of an ox (i.e., the name derives from the Latin term Taurus), which have gained great nutritional and pharmaceutical interest [190,191]. Taurine is an osmostress protectant in many marine metazoans and algae [192] and has also recently become a common component in beverages, foods and nutritional supplements [193] for its bioactive properties, including cardiovascular and antihypertensive effects [194].

### 2.9. Bioactive Polyketides and Macrolides

Polyketides and macrolides from marine dinoflagellates have been widely studied, especially those coming from strains of the dinoflagellate genus *Amphidinium* [195]. Little is known about the biosynthesis of such polyketides since dinoflagellates feature extraordinary big genome sizes [196], and the studies performed at the transcriptome level are insufficient to characterize the pathways involved in their biosynthesis even if some related transcripts can be detected [17,197]. Amphidinols (AMs) are a family of linear polyketides, the first compound of which was discovered 30 years ago [198]. All amphidinols discovered so far have been tested for their antifungal activity. For instance, amphidinol 2 and amphidinol 6 were found to be active against *Aspergillus niger* at 6 µg per disk [199]. Echigoya and co-workers observed strong antifungal activity on *A. niger* for AM2, AM4, and AM9 (44.3, 58.2 and 32.9 µg per disk, respectively), while the activity for AM10, AM11, AM12, and AM13 was rather low (>100–256.6 µg per disk) [200]. Amphidinol 18 displayed strong activity against the fungus *Candida albicans* (MIC 9 µg/mL) while the antifungal activity of its amphidinol 19 was absent [201].

Satake et al. [202] isolated and described the largest amphidinol homologues, amphidinol 20 and amphidinol 21, but they did not observe antifungal activity in *Aspergillus niger* even at the highest concentration tested (15 µg per disk). The antifungal activity of amphidinol 22 was also low, with a MIC value of 64 µg/mL for *C. albicans* and *A. fumigatus* [203] (Table 5).

**Figure 6:**
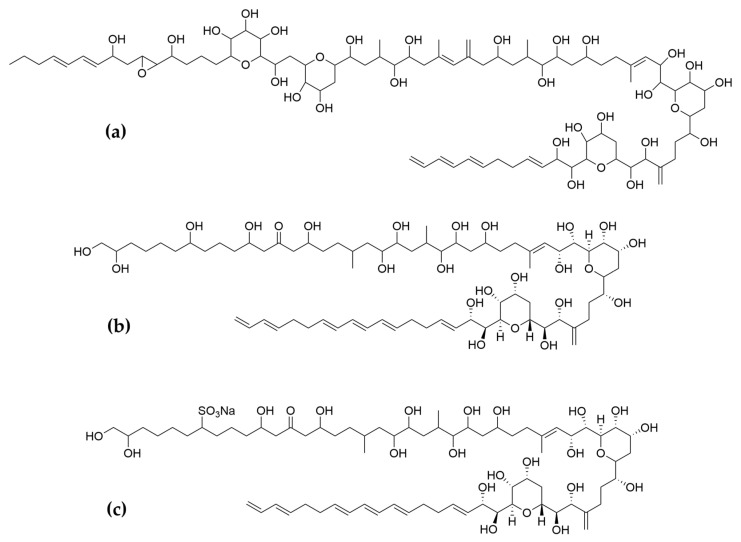
Examples of (**a**) long chain, amphidinol 22; (**b**) short chain, amphidinol 18; (**c**) sulfonated amphidinols, amphidinol 19.

Considering all these examples, the trend of the structure–bioactivity relationship indicates that amphidinols possessing longer chains and sulfonate derivates denoted weaker antifungal activities (Figure 6). The trend observed for their haemolytic activity on human erythrocytes is similar, as observed by Echigoya and co-workers [200]. Satake et al. [202] proposed membrane permeabilization (formation of channels) as the mode of action, and two different models to explain the difference in bioactivity of amphidinols. While short-chain amphidinols form a “spike” with a sterol molecule (such as cholesterol in human cells or ergosterol in fungi) in order to penetrate the membrane forming a barrel-stave channel, the long chain amphidinols are folded in a carpet-model bound to the lipidic portion of the membrane bilayer. These models also explain why amphidinols with voluminous polar substituents in the chain (as the sodium sulphate group OSO_3_Na^−^) do not display strong bioactivity, since such moieties will be repelled by the lipidic fraction of the bilayer.

**Figure 7:**
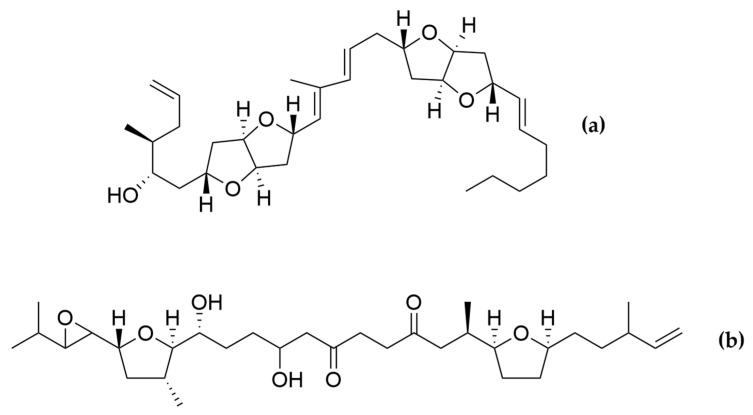
(**a**) Amphirionin-2 and (**b**) amphirionin-5. Two polyketides from the same family, but opposite bioactivities.

Two amphidinols have shown cytotoxic effects on cancer cells. For instance, amphidinol 2 displayed anticancer properties against HCT116 (colon carcinoma), HT-29 (colon adenocarcinoma) and MCF7 cancer cell lines (Table 5). After treatment with amphidinol 2, a 100-fold up-regulation of the early apoptotic markers cfos/cjun was also observed, suggesting apoptosis as the mechanism of action [205]. Amphidinol 22 displayed cytotoxic activity against lung cancer A549, melanoma A2058, liver cancer HepG2, breast cancer MCF7 and pancreas cancer MiaPaca2 cell lines, with IC_50_ values of 8 µM, 16.4 µM, 6.8 µM, 16.8 µM and 8.6 µM, respectively [203]. However, the mechanism of action for amphidinol 22 was not studied. Other polyketides such as the amphirionin-2 (Figure 7) have also displayed cytotoxic effects on cancer cells. Amphirionin-2 demonstrated potent cytotoxicity against colon cancer cell line Caco-2 and lung cancer cell line A549 [212]. However, the closely related compound amphirionin-5 (Figure 7) was found to promote the proliferation of cells instead of displaying cytotoxic activity [216]. This is an example of two compounds belonging structurally to the same family, but presenting completely opposite activities.

**Figure 8:**
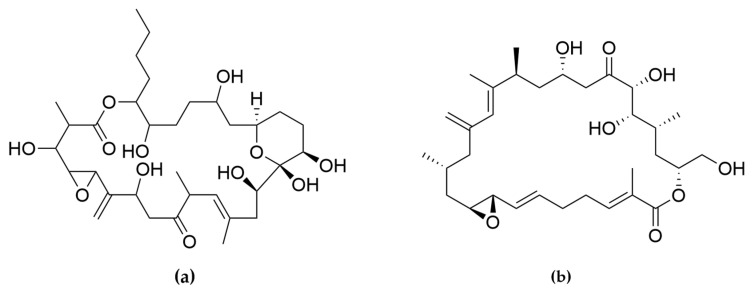
(**a**) Amphidinolides N; (**b**) Amphidinolides H. Amphidinolides with the highest cytotoxicity.

Amphidinolides are a family of cytotoxic macrolides isolated for the first time in the 80′s from dinoflagellates belonging to the genus *Amphidinium* [217]. From the more than 40 members which belong to this family of compounds, amphinolides N and H (Figure 8) exhibited the most potent activities, being extremely cytotoxic against L1210 murine leukemia cells (IC_50_ values of 0.05 and 0.48 ng/mL), and KB human epidermoid carcinoma cells (IC_50_ values of 0.06 and 0.52 ng/mL) [211]. The activity of amphidinolide H was explained by a covalent binding mechanism on the actin Tyr200 subdomain, stabilizing the actin filament [218]. On the other hand, amphidinolide N seems to have a higher affinity for the mitochondria of malignant cells rather than for the cytoskeletal structures [211].

#### Toxins with Potential Human Health Applications

Dinoflagellates are also able to produce a large diversity of metabolites, including biologically active compounds that are potentially toxic, [219] and are in fact often associated to harmful algal blooms, accounting for 75% of the species responsible for such phenomena [220]. Biosynthetic pathways for polyether ladder toxins (e.g., ciguatoxins, brevetoxins, maitotoxin, and yessotoxins) and linear polyether toxins (okadaic acid and dinophysistoxins) have been studied, and their biosynthesis involves modular polyketide and non-ribosomal peptide mega-synthetases able to catalyse processes such as polyepoxide cascades, Favorskii-like rearrangements, acetate C1 carbon deletions, consecutive acetate additions to a starter glycolate, Baeyer-Villiger oxidations, side-chain acetate replacements with glycine and aldol condensations between a backbone carbonyl and acetate or malonate [221]. In humans, exposure to these toxins can lead to gastrointestinal and neurological syndromes (i.e., paralytic shellfish poisoning—PSP, amnesic shellfish poisoning—ASP, diarrheic shellfish poisoning—DSP, neurologic shellfish poisoning—NSP, and ciguatera fish poisoning—CFP) and even death [219]. However, several publications have reported microalgal toxins as displaying important biological activities, which are or could be of interest for possible human health applications (Table 6).

Most of the compounds present in Table 6 are known to induce harmful effects on humans due to alterations on voltage-gated channels (sodium, potassium or calcium) of human cells. Using a specific range of concentrations (safe windows) or derivatising toxic active principles are two common approaches to avoid toxicity in active principles when it comes to avoid failure during clinical trials and successfully develop drugs [236]. This is the reason why toxins from marine microalgae should not be discarded as active principles for potential future drugs.

**Figure 9:**
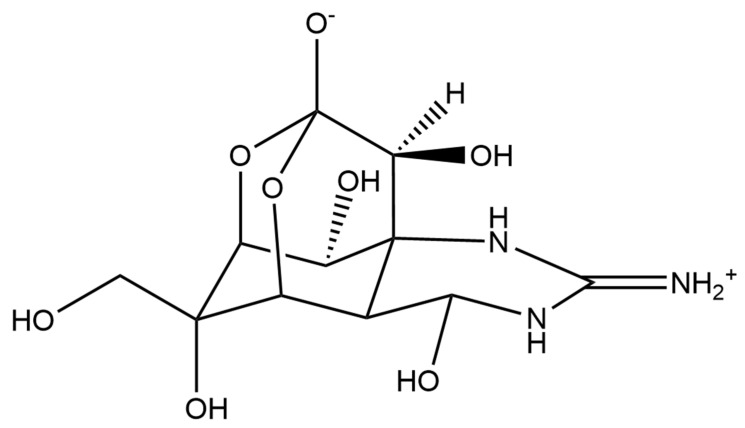
Bidimensional structure of tetrodotoxin.

For examples Halneuron^®^ is a pain medication undergoing phase 3 clinical trials for the treatment of chemotherapy-induced neuropathic pain on patients with cancer. Its active principle is tetrodotoxin (Figure 9), which was also found in the dinoflagellate *Alexandrium tamarense* [237] known to induce damage on skeletal muscle tissue and peripheral nerves, being the major cause of intoxication and respiratory failure. These effects are observed due to the mechanisms of the toxin, which blocks the influx of sodium ions in voltage-gated sodium channels [238]. However, using the adequate doses, tetrodotoxin has been shown to possess beneficial effects against acute, inflammatory and neuropathic pain in animal models [224]. Clinical trials are also reported in the literature. The studies performed consisted in multi-centre, randomized, double-blind, placebo-controlled, parallel-designed trials to test the efficacy and safety of TTX on individuals older than 18 years to severe cancer-related pain. The results found clinically relevant analgesic effects on cancer-related pain with a favourable benefit-risk profile [225]. According to Wex Pharmaceuticals Inc., its pain medicine Halneuron^®^ has been tested on more than 500 patients and showed evidences of long duration of pain relief with minimal side effects (https://wexpharma.com/; accessed on 21 April 2021).

**Figure 10:**
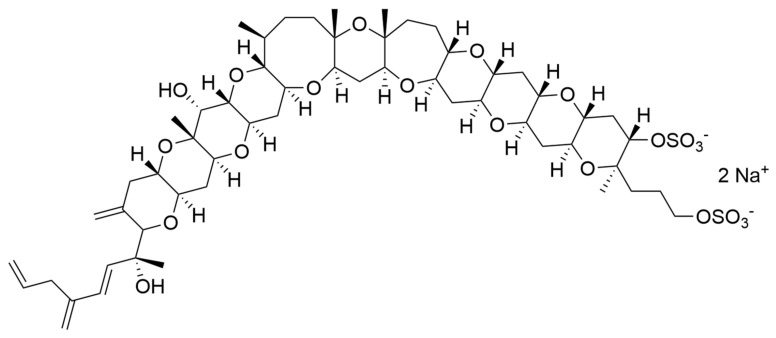
Bidimensional structure of yessotoxin.

Another example of a toxin with possible pharmaceutical applications is yessotoxin (Figure 10), a shellfish biotoxin responsible for diarrheic shellfish poisoning (DSP) and found in the dinoflagellate *Protoceratium reticulatum* [239]. A potent long-term neurotoxic effect was also reported in mice cerebellar neurons when they were exposed to yessotoxin at concentrations as low as 25 nM. A Spanish patent reported the use of yessotoxin and its derivatives on prevention of neurological diseases related to abnormal levels of tau and β-amyloid proteins. At a concentration of 1 nM, it reduced the levels of intracellular β-amyloid in cells of triple-transgenic mouse model of Alzheimer disease (3xTg-AD). It also considerably reduced the hyperphosphorylation of tau [229]. In addition to anti-Alzheimer tests, the antiproliferative effect on cancer cells was largely studied and the molecule was found to restrain cell growth in several cancer cells (Table 6). The anti-allergic effect of the molecule was also tested, but the results showed minor effects [228].

**Figure 11:**
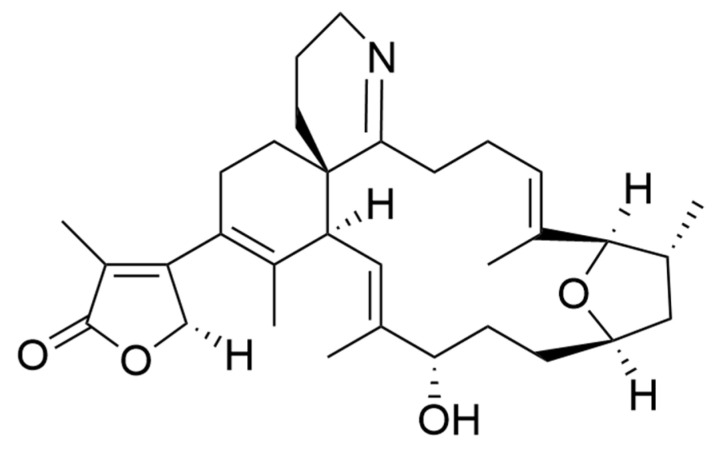
Bidimensional structure of gymnodimine.

Gymnodimine (Figure 11) produced by the dinoflagellate *Gymnodinium* sp. [240] is a toxin that also presented anti-Alzheimer properties in vitro, reducing intracellular amyloid-beta levels and tau hyperphosphorylation (Table 6). However, the concentration needed was higher compare to yessotoxin (50 nM compared to 1 nM in the case of yessotoxin).

**Figure 12:**
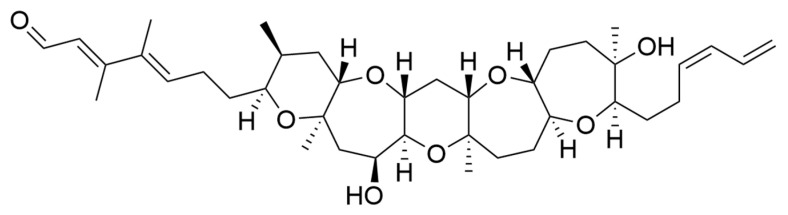
Bidimensional structure of brevenal.

Brevetoxins are potent marine neurotoxins closely related to ciguatoxins and associated to gastrointestinal, neurological and cardiovascular harmful effects on humans [241,242]. However, brevenal (Figure 12) is one member of the family of the brevetoxins that have demonstrated low in vitro toxicity against human/murine cell lines and potent anti-inflammatory effects on adenocarcinoma cell line A549 and murine macrophages RAW 264.7 (Table 6). Brevenal reduced the lipopolysaccharide (LPS)-induced production of the pro-inflammatory chemokine IL-8 (A549) at the nanomolar level and the production of the pro-inflammatory cytokine TNF-α (RAW 264.7) at the picomolar level. Such findings suggest the unexploited potential of brevenal for applications on pulmonary diseases [234].

In general, the most important applications of toxins coming from marine dinoflagellates are oriented to the fields of pain relief and neurological disorders related to β-amyloid accumulation and tau hyperphosphorylation. Some toxins have also displayed antifungal and antiproliferative (cancer cells) properties, but such activities seem to be rather unspecific. For instance, okadaic acid showed antifungal activity [213] but it is known to display cytotoxicity, neurotoxicity, immunotoxicity, embryotoxicity, and tumour promoting properties as well [243].

## 3. Discussion

Microalgae have recently gained a lot of attention due to the production of high-added value products with different possible health applications [244]. However, the cell targets of these compounds and their mechanism of action are often completely unknown and further research is necessary to unlock the biotechnological potential of these metabolites. Importantly, various microalgae (e.g., *Chlorella* and *Dunaliella*) have generally received the safe (GRAS) status, which implies that, according to the U.S. food and drug administration (FDA), they are “safe to consume” [245].

The number of species for which the genome is available or that have been successfully genetically modified remains extremely low. More genome sequencing, bioactivity screening, and species transformations are needed, especially for microalgae of commercial value.

The routine use of transgenic microalgae for the production of marketable products (e.g., carotenoids, fatty acids, biofuel, vaccines and bioactives) is a great challenge for the coming years. At present, highly advanced industrial biotechnology systems using bacteria (e.g., *Escherichia coli*, *Bacillus subtilis*, *Corynebacterium glutamicum*, *Lactobacillus* spp.), and yeasts and fungi (e.g., *Saccharomyces cerevisiae*, *Aspergillus* spp.) are in use as classical metabolic engineering systems. Encouraging examples are the report of transgenic microalgae with enhanced ability to bind heavy metals by using a foreign metallothionein [246] for bioremediation, the biological production of hydrogen by a genetically modified *Chlamydomonas* clone for biofuel generation, the production of human erythropoietin, fibrinectin, interferon β1, proinsulin, vascular endothelial growth factor and high mobility group protein B1 by *Chlamydomonas* with potential pharmaceutical applications [247], and the implemented production of carotenoids and lipids for different applications [244,246,248]. Microalgae are considered to be an outstanding candidate for biomass production (nearly 77% of dry cell mass), photosynthesis processes for lipid fabrication, and the production of biofuel [249,250].

Considering the physicochemical limitations and technological challenges reported for the incorporation of bioactives into products, namely high instability, poor aqueous solubility, and low bioavailability, encapsulation systems appear as an emerging and significant tool to overcome such issues. Microalgae bioactives can have applications in several areas, but, in some cases, without proper protection during processing and storage, as well as, without suitable biopharmaceutical properties, the efficacy of their functionality may be absolutely compromised. For this reason, microalgae encapsulation is another intensively investigated field [251]. Many of the systems developed in this regard have proven their effectiveness in terms of stability and bioavailability improvement, suggesting they could also be applied for pharmaceutical or cosmetic purposes after in vitro and in vivo biological activity determination. On the market, there are only few examples of commercial products that claim to contain encapsulated microalgae bioactives, particularly products based on astaxanthin from *H. pluvialis* [252] and carotenoids from the microalga *D. salina* [253].

Different approaches have been used to implement the production of compounds of interest, from culturing condition optimization, adaptive laboratory evolution (ALE), mutagenesis, and genetic engineering techniques [3,254]. Large-scale production by microalgae is more feasible compared to marine macroorganisms or terrestrial plants. Microalgae are amenable to culturing, requiring few nutrients and light, in eco-friendly and eco-sustainable manner, without negative impacts on the environment. However, costs are still high and productivity is sometimes very low. New technologies (e.g., finely regulated culturing in tubular, flat plate, twin-layers, inclined tubular, helical and column photobioreactors; [255,256]) are focused on implementing production and reducing costs. In addition, system biology and synthetic biology will give a great boost to this fast-growing sector, offering further opportunities for producing active ingredients for different biotechnological applications in pharmaceutical, nutraceutical, and cosmeceutical sectors.

## Figures and Tables

**Table 5:** This table includes the name of the different polyketides and macrolides, the dinoflagellates from which they were originally isolated, their biological activity, the active concentration for the different assays and the references.

Compound	Microalgae	Bioactivity	Concentration	Ref.
Amphidinols 1, 2, 4, 5 and 6 (AM1, AM2, AM4, AM5, AM6)	*Amphidinium klebsii*	Antifungal activity against *Aspergillus niger* Haemolytic activity on human erythrocytes	In vitro disk assay: Minimum effect concentration (MEC) value 4 to 6 µg/disk In vitro Half maximum effective concentrations (EC_50_ values): 50 nM (AM1) 910 nM (AM2) 185 nM (AM4) 230 nM (AM5) 580 nM (AM6)	[204]
Amphidinol 2 (AM2)	*Amphidinium klebsii*	Anticancer activity against colon cancer cells HCT116, HT29, and breast cancer cells MCF7	Half maximal inhibitory concentration (IC_50_) values: 6.18 µM (HCT116) 0.87 µM (HT29) 2.98 µM (MCF7)	[205]
Amphidinol 3 (AM3)	*Amphidinium klebsii*	Antifungal activity against *Aspergillus niger* Haemolytic activity on human erythrocytes	In vitro disk assay: MEC value 9 µg/disk In vitro EC_50_ value: 250 nM	[206]
Amphidinol 7 (AM7)	*Amphidinium klebsii*	Antifungal activity against *Aspergillus niger* Haemolytic activity on human erythrocytes	In vitro disk assay: MEC value 10 µg/disk In vitro EC_50_ value: 300 nM	[207]
Amphidinol 9 (AM9)	*Amphidinium carterae*	Antifungal activity against *Aspergillus niger* Haemolytic activity on human erythrocytes	In vitro disk assay: MEC value 10 µg/disk In vitro EC_50_ value: 300 nM	[200]
Amphidinols 10, 11, 12 and 13 (AM10, AM11, AM12 and AM13)	*Amphidinium carterae*	Antifungal activity against *Aspergillus niger* Haemolytic activity on human erythrocytes	In vitro disk assay: MEC value >100 µg/disk In vitro EC_50_ value: >2000 nM	[200]
Amphidinols 14 and 15 (AM14 and AM15)	*Amphidinium klebsii*	Antifungal activity against *Aspergillus niger* Haemolytic activity on human erythrocytes	In vitro disk assay: MEC value >60 µg/disk In vitro EC_50_ value: >50 µM	[199]
Amphidinol 17 (AM17)	*Amphidinium carterae*	Antifungal activity against *Aspergillus niger*, and *Candida kefyr*. Haemolytic activity on human erythrocytes	In vitro disk assay: not detectable antifungal activity In vitro EC_50_ value: >4.5 µM	[208]
Amphidinols 18 and 19 (AM18 and AM19)	*Amphidinium carterae*	Antifungal activity against *Candida albicans*	In vitro growth inhibition assay: MIC values 9 µg/mL (AM18) Not detectable activity (AM19)	[201]
Amphidinols 20 and 21 (AM20 and AM21)	*Amphidinium carterae*	Antifungal activity against *Aspergillus niger* Haemolytic activity on human erythrocytes	In vitro disk assay: MEC value >15 µg/disk In vitro EC_50_ value: 1–3 µM (AM20) >10 µM (AM21)	[202]
Amphidinol 22 (AM22)	*Amphidinium carterae*	Antifugal activity against *Candida albicans* and *Aspergillus fumigatus* Anticancer activity against several cancer cell lines	Minimum inhibitory concentration value (MIC): 64 µg/mL In vitro: Half maximal inhibitory concentration (IC_50_) values: from 6 to 16 µM	[203]
Amphidinols A and B (AM-A and AM-B)	*Amphidinium carterae*	Antifungal activity against *Candida albicans*	In vitro growth inhibition assay: MIC values 19 µg/mL (AM-A) >150 µg/mL (AM-B)	[209]
Karantungiol A	*Amphidinium* sp.	Antifungal activity against NBRC4407 *Aspergillus niger* Antiprotozoal activity against *Trichomonas* *foetus*	In vitro disk assay: 12 µg/disc In vitro Antiprotozoal assay: MIC 1 µg/mL	[210]
Amphidinolides H and N (AMP-H and AMP-N)	*Amphidinium* sp.	Anticancer activity against L1210 murine leukemia cells and KB human epidermoid carcinoma cells	Half maximal inhibitory concentration (IC_50_) values: 0.48 ng/mL (AMP-H, L1210) 0.52 ng/mL (AMP-H, KB) 0.05 ng/mL (AMP-N, L1210) 0.06 ng/mL (AMP-N, KB)	[211]
Amphirionin 2	*Amphidinium* sp.	Anticancer activity against colon cancer Caco-2 and lung cancer A549 cells	Half maximal inhibitory concentration (IC_50_) values: 100 ng/mL (Caco-2) 600 ng/mL (A549)	[212]
Gambieric acids A and B	*Gambierdiscus toxicus*	Antifungal activity against several fungal strains	In vitro growth inhibition assay: MIC values 0.2 to 6.25 µg/mL depending on compound/strain	[213]
Goniodomin A	*Goniodoma pseudogoniaulax*	Antifungal activity against *Mortierella ramannianus* and *Candida albicans* Reduction on metabolic rate of BE(2)-M17 human neuroblastoma cells	Growth inhibition at a concentration of 0.5 µg/mL In vitro: 50% decreased metabolic rate after 6-h incubation with 15 μM	[214,215]

**Table 6:** This table includes the name of the different toxins, the dinoflagellates from which they were originally isolated, their biological activity, the active concentration for the different assays and bibliographic references.

Compound	Microalgae	Bioactivity	Concentration/dosing	Ref.
Saxitoxin	*Alexandrium* sp.	Local anesthetic (Rat sciatic nerve)	In vivo: 58 ± 3 nmol/mL (for 60 min analgesia)	[222]
Neosaxitoxin	*Alexandrium* sp.	Local anesthetic (Rat sciatic nerve) Bladder pain blocker (Human)	In vivo: 34 ± 2 nmol/mL (for 60 min analgesia) In vivo: dose of 80 µg, with successful prolonged pain reduction in all the patients.	[222]
Gonyautoxins 2/3	*Alexandrium* sp.	Chronic headache (Human)	In vivo: dose of 50 µg (70% of the patients responded, long lasting effect)	[223]
Tetrodotoxin	*Alexandrium tamarense*	Acute, inflammatory and neuropathic pain (animal models) Several to moderate cancer-related pain (human)	In vivo: several different doses/ application methods In vivo: eight doses of 30 µg (during 4 days) for prolonged pain relief	[224,225]
Okadaic acid	*Porocentrum lima*	Antifungal activity on *Aspergillus niger* and *Penicillium funiculosum*	Disc test: 10 µg/disc (inhibition circle observed)	[213]
Yessotoxin	*Protoceratium reticulatum*	Cell death inducer in several cancer cell lines (27 out of 58 cell lines) Antiproliferative activity on BC3H1 myoblast cells Antiproliferative activity on RBL-2H3 and B16F10 melanoma cells Anti-Alzheimer activity	In vitro: nanomolar scale concentrations, different mechanisms In vitro: 100 nM induced autophagy in BC3H1 cells In vitro: 10–100 nM drastically reduced cell viability by apoptotic cell death In vitro: 1 nM reduced β-amyloid deposition and Tau protein hyperphosphorylation	[226,227,228,229]
Pectenotoxin 2	*Dinophysis* sp.	Antiproliferative activity of several cancer cell lines, particularly strong for COLO205 colon cancer cells.	In vitro: LC_50_ value of 8 nM for COLO205	[230]
Maitotoxin 3	*Gambierdiscus belizeanus*	Antiproliferative activity on SH-SY5Y human neuroblastoma cells	In vitro: IC_50_ value of 0.7 µM	[231]
Gambierol	*Gambierdiscus toxicus*	Immunomodulatory activity on immune cells expressing K^+^ channels	In vitro K_v_1.1–1.5 channel inhibition: IC_50_ values at the nanomolar scale	[232]
Brevetoxins 2, 3, 6, 9	*Karenia brevis*	Antiproliferative activity on leukemic cells Jurkat E6-1	In vitro: from 5 to 60 mM. Brevetoxin 2 was the most potent with IC_50_ 5.6 mM	[233]
Brevenal	*Karenia brevis*	Anti-Inflammatory activity on adenocarcinoma cell line A549 Anti-Inflammatory activity on murine macrophages RAW 264.7	0.1 and 1 nM reduced the level of pro-inflammatory chemokine IL-8 0.1 and 1 pM reduced the level of pro-inflammatory cytokine TNF-α	[234]
Gymnodimine	*Gymnodinium* sp.	Anti-Alzheimer activity by reduction on intracellular amyloid-beta levels and reduction of tau hyperphosphorylation	50 nM reduced β-amyloid expression by 20.9 ± 0.6% (3–7 days in culture) and decreased tau hyperphosphorylation by 34–37%	[235]
